# Efficacy and safety of new-generation Bruton tyrosine kinase inhibitors in chronic lymphocytic leukemia/small lymphocytic lymphoma: a systematic review and meta-analysis

**DOI:** 10.1007/s00277-023-05486-x

**Published:** 2023-10-16

**Authors:** Shuo Yin, Xiaohong Zheng, Weichunbai Zhang, Hanyun Zhao, Rong Zhang, Wenbin Li, Feng Chen

**Affiliations:** Department of Neuro-Oncology, Cancer Center, Beijing Tiantan Hospital, Capital Medical University, Beijing, 100070 China

**Keywords:** Chronic lymphocytic leukemia, Small lymphocytic lymphoma, Bruton tyrosine kinase inhibitor, Systematic review, Meta-analysis

## Abstract

Chronic lymphocytic leukemia/small lymphocytic lymphoma (CLL/SLL) is a type of mature B lymphocyte clonal proliferative tumor with a specific immunophenotype. Bruton tyrosine kinase inhibitors (BTKi) have been approved for the treatment of CLL/SLL. However, the efficacy and safety of new-generation BTKi-based regimens have not been systematically studied. In this systematic review, we evaluated the efficacy and safety of new-generation BTKi-based regimens for the treatment of patients with CLL/SLL. A comprehensive search on PubMed, Embase, Cochrane Library, and ClinicalTrials.gov. up to January 31, 2023, was conducted by us. Studies reporting data on CLL/SLL patients treated with new-generation BTKi were included. We assessed the overall response rate (ORR), complete response (CR) rate, and 24-month OS/PFS rates for efficacy analysis. For safety analysis, we evaluated the incidence of grade ≥ 3 adverse events (AEs). The meta-analysis included twenty studies. The pooled ORR for new-generation BTKi was 92% (95% CI, 89–95%, *I*^*2*^ = 80.68%, *P* = 0.00), while the pooled CR rate was 10% (95% CI, 6–14%, *I*^*2*^ = 88.11%, *P* = 0.00). Research has found that the new-generation BTKi-based therapy had higher efficacy under the following treatment conditions: < 65 years old, treatment-naive (TN)-CLL, and BTKi combination therapy. The ORR/CR rates and 24-month OS/PFS rates of BTKi combination therapy were higher than that of BTKi monotherapy. Compared to acalabrutinib monotherapy, zanubrutinib monotherapy demonstrated higher ORR/CR rates and 24-month OS/PFS rates. Common grade ≥ 3 AEs included cytopenia and hypertension. The new-generation BTKi-based therapy has good tolerance and provides incremental benefits for CLL/SLL patients. Despite the superior efficacy of BTKi combination therapy compared to monotherapy, its AEs rates are relatively high. Compared to acalabrutinib, Zanubrutinib may be the preferred monotherapy for CLL. However, randomized-controlled studies are still needed.

**Supplementary Information:**

The online version contains supplementary material available at 10.1007/s00277-023-05486-x.

## Introduction

Chronic lymphocytic leukemia/small lymphocytic lymphoma (CLL/SLL) is a kind of mature B-lymphocyte clonal proliferative tumor characterized by a specific immunophenotype. It is characterized by the accumulation of lymphocytes in peripheral blood, bone marrow, lymph nodes, and spleen, and primarily affects middle-aged and elderly individuals ^[1]^. As the most common leukemia in adults in Western countries, CLL accounts for approximately 25–35% of all leukemias in the USA. This disease is more common among white Americans and is predominantly male. The median age at diagnosis is approximately 72 years old. In 2023, an estimated 18,740 people will be diagnosed with CLL/SLL in the USA, and an estimated 4490 people will die from this disease^[2,3]^. CLL is a highly heterogeneous disease. Over the past two decades, a major focus of pharmacologic research was signaling through the B-cell receptor (BCR). Several BCR-targeted agents have been approved for use in CLL patients, including Bruton tyrosine kinase (BTK) inhibitors.

In the USA, the Food and Drug Administration (FDA) has approved Ibrutinib, which is a first-in-class BTKi that irreversibly binds to BTK, for the treatment of CLL.^[4]^. Ibrutinib offers a chemotherapy-free treatment option initially explored in relapsed or refractory (R/R) CLL/SLL patients. The PCYC-1103 study established 420 mg as the RP2D and the RESONATE trial proved that ibrutinib is superior to anti-CD20 ofatumumab^[5,6]^. With continued research, treatment-related adverse events such as bleeding, diarrhea, and cardiovascular toxicity have attracted attention. Binding of ibrutinib to additional kinases (e.g., EGFR TEC ITK) may potentially contribute to these side effects, prompting development of new-generation BTKi that demonstrate more specific and sustained effects compared to first-generation BTKi in in vivo and preclinical models ^[7]^. The new-generation BTKi currently used to treat CLL include acalabrutinib, zanubrutinib, and tirabrutinib which have higher BTK selectivity and lower/no inhibitory effect on EGFR, TEC, ITK, etc.

We conducted a comprehensive search of CLL studies related to next-generation BTKi treatment in this meta-analysis study and analyzed efficacy and safety data. In addition, we further evaluated the efficacy of BTKi treatment through subgroup analysis, aiming to provide compelling evidence for more rational and effective application of BTKi.

## Methods

### Registration and protocol

The systematic review protocol was registered on the PROSPERO under CRD42023398266.

### Search strategy

We carefully searched PubMed, Embase, the Cochrane Library, and ClinicalTrials.gov to identify relevant studies published up to January 31, 2023, using subject words combined with free words. The subject words were “Leukemia, Lymphocytic, Chronic, B-Cell” and “Bruton Tyrosine Kinase Inhibitor, acalabrutinib, zanubrutinib, orelabrutinib, tirabrutinib”; Supplementary Table S1 provides detailed search terms used in different databases. To ensure a comprehensive search and evaluation of all potentially relevant studies, the search will not be limited by region, race, or age. In addition, the list of references for the identified articles and comments has been rigorously checked by us.

### Inclusion and exclusion criteria

The inclusion criteria: (1) prospective clinical studies (including single-arm studies and randomized control trials); (2) studies including patients diagnosed with CLL/SLL; (3) studies involving patients treated with new-generation BTKi (acalabrutinib, zanubrutinib, orelabrutinib, or tirabrutinib), both as single-agent therapy and in combination with other agents; (4) studies reporting both efficacy and safety endpoints, including the overall response rate (ORR), complete response (CR) rate, and adverse events (AEs); (5) published in English and related to human clinical trial.

The exclusion criteria: (1) differences in the doses assigned to patients; (2) significant flaws in statistical methods or experimental design; (3) the repeated publication or similar research; (4) reported outcomes treated by ibrutinib; (5) article type: conference abstract, comment, letters, review, and case report; (6) reported incomplete information; (7) cell or animal study.

### Data extraction

All relevant studies were imported into Endnote 9.1 software, and duplicates were subsequently removed. Two reviewers independently extract duplicate data based on inclusion and exclusion criteria (YS, ZXH) using a self-designed data collection form. In case of any discrepancies or disagreements, by consulting the third author (CF) or consensus-based discussion.

The following information was extracted from each study: (1) general characteristics of each study (first author’s name, publication year, ClinicalTrials.gov Identifier, phase of the study); (2) descriptive data of the patients (number of patients, median age, disease status); (3) treatment strategies and the dose of BTKi; (4) primary efficacy endpoints (ORR, CRR) and secondary efficacy endpoints (24-month OS/PFS rate); (5) the number of grade ≥ 3 AEs.

### Study outcome evaluation

The definition of ORR was the proportion of patients with CR, CR with incomplete hematological recovery, nodular partial response (PR), PR with lymphocytosis, or PR. The CR rate included CR and eliminated CR with incomplete hematological recovery. Response assessments were conducted for CLL per the “International Workshop on Chronic Lymphocytic Leukemia” (IWCLL) 2008 criteria^[8]^, and for SLL per the Lugano classification for lymphoma 2014^[9]^. Besides, “the National Cancer Institute Common Terminology Criteria for Adverse Events, version 4.03” will be used to classify different types of AEs. PFS was defined as the time from the randomization date to progressive disease or death from any cause, and OS was calculated as the date from the random assignment until death due to any cause.

### Quality and risk of bias assessment

The methodological quality and risk of bias of included randomized controlled trials were assessed using the Cochrane risk-of-bias tool^[10]^. The methodological index for non-randomized studies (MINORS) was used to assess prospective single-arm studies.^[11]^.

### Statistical analysis

All data analyses were performed using the STATA SE15.1 (StataCorp, TX, USA). For new-generation BTKi-based regime efficacy, we calculated the pooled ORR and CR rate, with 95% confidence intervals (CI). The Cochrane’s Q chi-square test and *I*^2^ statistic were used to examine the heterogeneity across studies. *P* < 0.05 was considered statistically significant. The fixed-effects model was used for pooled results with low heterogeneity (*I*^2^ ≤ 50% and/or *P* ≥ 0.10); otherwise, the random-effects model was used for analysis. By excluding each study one by one from the pooled results with high heterogeneity, sensitivity analysis was performed. To explore the potential impact of different factors on the measurement of results, the sub-group analysis will be conducted on variables including age (< 65 vs. ≥ 65 years), disease status (TN-CLL vs. RR-CLL), treatment strategy (monotherapy vs. combination therapy), monotherapy (acalabrutinib vs. zanubrutinib). For safety, we calculated the toxicity rate similarly, with 95% confidence intervals, and the subgroup analysis by monotherapy was applied. Publication bias was assessed using Begg’s and Egger’s tests. Significant publication bias was defined as a *P* value < 0.05.

## Results

### Study selection and characteristics

**Fig. 1:**
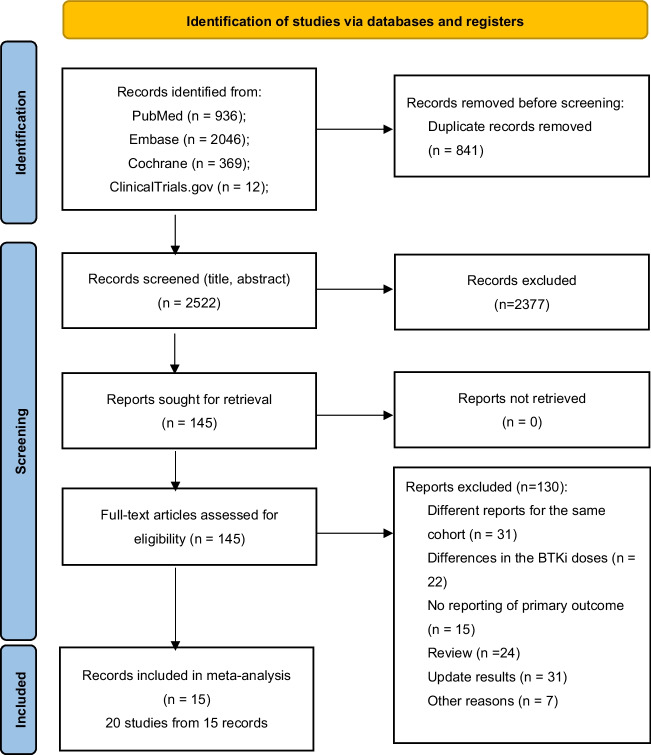
Flow diagram of study selection

In our search, a total of 3363 records were retrieved, including 841 duplicate reports and 2522 records that underwent title and abstract review. A total of 2377 records were excluded due to the following reasons: other diseases (*n* = 41), other drugs (*n* = 361), conference abstract (*n* = 1278), case reports (*n* = 21), reviews (*n* = 533), meta-analyses (*n* = 8), note (*n* = 29), letter (*n* = 24), cell/animal studies (*n* = 24), and National Clinical Trial registration (*n* = 58). For 145 records, the full text was reviewed, and 130 of them were excluded based on following reasons: different reports for the same cohort (*n* = 31), review (*n* = 24), updated results (*n* = 31), no reporting of the primary outcome (*n* = 15), differences in the BTKi doses (*n* = 22) and other reasons (*n* = 7). Ultimately, our meta-analysis included 15 records. Figure 1 showed the literature and identification process. The meta-analysis evaluated the efficacy and safety of the new-generation BTKi for a total of 2066 CLL/SLL patients, across ten single-arm studies and five randomized studies. Considering that some records have more than one disease state or intervention, we divide them into 20 studies. Table 1 summarized the baseline clinical characteristics of these patients.

**Table 1:** Basic characteristics of the included studies

No	Study	NCT	Study type	Disease status	Intervention	Sample size	Age	Median follow-up (months)	Reported outcomes	
1	Ghia,P 2022^[12]^	NCT02970318	III	RR-CLL	Acalabrutinib (100 mg) twice daily	155	68 (32–89)	46.5	OR, CR	AEs
2	Byrd, J. C 2021^[13]^	NCT02029443	I/II	TN-CLL	Acalabrutinib (100 mg) twice daily	99	64 (33–85)	53	OR, CR	AEs
3	Awan, F. T 2019^[14]^	NCT02029443	I/II	Ibrutinib-intolerant CLL	Acalabrutinib (100 mg) twice daily	35	64 (50–82)	Safety: 18.5 Efficacy: 19	OR, CR, 24-PFS	AEs
4	Woyach, J. A 2020^[15]^	NCT02296918	I/II	TN-CLL	Acalabrutinib (100 mg) twice daily + obinutuzumab	19	61 (42–75)	39 (range, 1–45)	OR, CR, 24-OS, 24-PFS	AEs
5				RR-CLL	Acalabrutinib (100 mg) twice daily + obinutuzumab	26	63 (42–76)	42 (range, 20–49)	OR, CR	
6	Byrd, J. C 2021^[16]^	NCT02477696	III	Previously treated-CLL	Acalabrutinib (100 mg) twice daily	268	66 (41–89)	40.9	OR, CR	AEs
7	Sharman,J.P 2020^[17]^	NCT02475681	III	TN-CLL	Acalabrutinib (100 mg) twice daily + obinutuzumab	179	70	28.3 (IQR 25.6–33.1)	OR, CR, 24-OS, 24-PFS	AEs
8				TN-CLL	Acalabrutinib (100 mg) twice daily	179	70	28.3 (IQR 25.6–33.1)	OR, CR, 24-OS, 24-PFS	AEs
9	Davids, M. S 2021^[18]^	NCT03580928	II	TN-CLL	Acalabrutinib (100 mg twice daily) + venetoclax + obinutuzumab	37	63	27.6 (IQR 25.1–28.2)	OR, CR	AEs
10	Rogers, K. A 2021^[19]^	NCT02717611	II	Ibrutinib intolerant R/R CLL	Acalabrutinib (100 mg) twice daily	60	69.5 (43–88)	35	OR, CR, 24-OS, 24-PFS	AEs
11	Sun, C 2020^[20]^	NCT02337829	II	TN/RR CLL	Acalabrutinib (100 mg) twice daily	24	64 (45–83)	-	OR, CR, 24-PFS	AEs
12				TN/RR CLL	Acalabrutinib (200 mg) once daily	24		-	OR, CR, 24-PFS	
13	Soumerai,J.D 2021^[21]^	NCT03824483	II	Previously untreated CLL	Zanubrutinib 160 mg twice daily	39	62 (52–70)	25.8 (IQR 24.0–27.3)	OR, CR	AEs
14	Xu, W 2020^[22]^	NCT03206918	II	RR-CLL	Zanubrutinib 160 mg twice daily	91	61 (35–87)	15.1	OR, CR	AEs
15	Tam, C. S 2022^[23]^	NCT03336333	III	TN CLL without del(17)(p13·1)	Zanubrutinib 160 mg twice daily	241	70 (66–75)	26.2 (IQR 23.7–29.6)	OR, CR, 24-OS, 24-PFS	AEs
16				TN CLL with del(17)(p13·1)	Zanubrutinib 160 mg twice daily	111	70 (66–74)	26.2 (IQR 23.7–29.6)	OR, CR, 24-OS, 24-PFS	AEs
17	Cull,G 2022^[24]^	NCT02343120	I/II	TN-CLL	Zanubrutinib 160 mg twice daily or 320 qd	22	69.5 (48–87)	47.2	OR, CR, 24-OS, 24-PFS	AEs
18				RR-CLL	Zanubrutinib 160 mg twice daily or 320 qd	101	66 (24–87)	47.2		
19	Brown, J. R. 2023^[25]^	NCT03734016	III	RR-CLL	Zanubrutinib 160 mg twice daily	327	67 (35–90)	29.6	OR, CR, 24-PFS	AEs
20	Danilov, A. V 2020^[26]^	NCT02457598	Ib	Previously treated CLL	Tirabrutinib 80 mg every day	29	70 (52–91)	-	OR, CR	AEs

*TN* treatment-naive, *RR* relapsed or refractory, *CLL* chronic lymphocytic leukemia, *OR* overall response, *CR* complete response, *OS* overall survival, *PFS* progression-free survival, *AEs* adverse events

### Quality assessment

Ten single-arm studies assessed using the MINORS index score ranged from 12 to 22 points, which was acceptable for the present meta-analysis **[**Table 2**]**. Five RCTs were independently evaluated for quality using the Cochrane Collaboration risk of bias tool **[**Supplementary Figure S1**]**.

**Table 2:** Quality assessment of included non-randomized studies

MINORS index for included non-randomized studies													
Study	I	II	III	IV	V	VI	VII	VIII	IX	X	XI	XII	Total
Byrd, J. C 2021	2	2	2	2	1	2	2	2	-	-	-	-	15
Awan, F. T 2019	2	2	2	2	1	1	2	0	-	-	-	-	12
Woyach, J. A 2020	2	2	2	2	1	2	2	2	2	2	1	2	22
Davids, M. S 2021	2	2	2	2	2	1	2	2	-	-	-	-	15
Rogers, K. A 2021	2	2	2	2	1	2	2	2	-	-	-	-	15
Sun, C 2020	2	2	2	2	1	1	2	2	2	2	2	2	22
Soumerai,J.D 2021	2	2	2	2	1	1	2	1	-	-	-	-	13
Xu, W 2020	2	2	2	2	2	1	2	2	-	-	-	-	15
Cull,G 2022	2	2	2	2	2	2	2	1	1	2	1	2	21
Danilov, A. V 2020	2	2	2	2	1	1	2	1	1	2	1	2	19

Numbers I-VIII in heading signified: I, a clearly stated aim; II, the inclusion of consecutive patients; III, prospective collection of data; IV, endpoints appropriate to the aim of the study; V, unbiased assessment of the study endpoint; VI, follow-up period appropriate to the aim of the study; VII, loss of follow up less than 5%; VIII, prospective calculation of the study size; IX, an adequate control group; X, contemporary groups; XI, baseline equivalence of groups; XII, adequate statistical analyses; -: none

### Efficacy

#### Tumor response

**Fig. 2:**
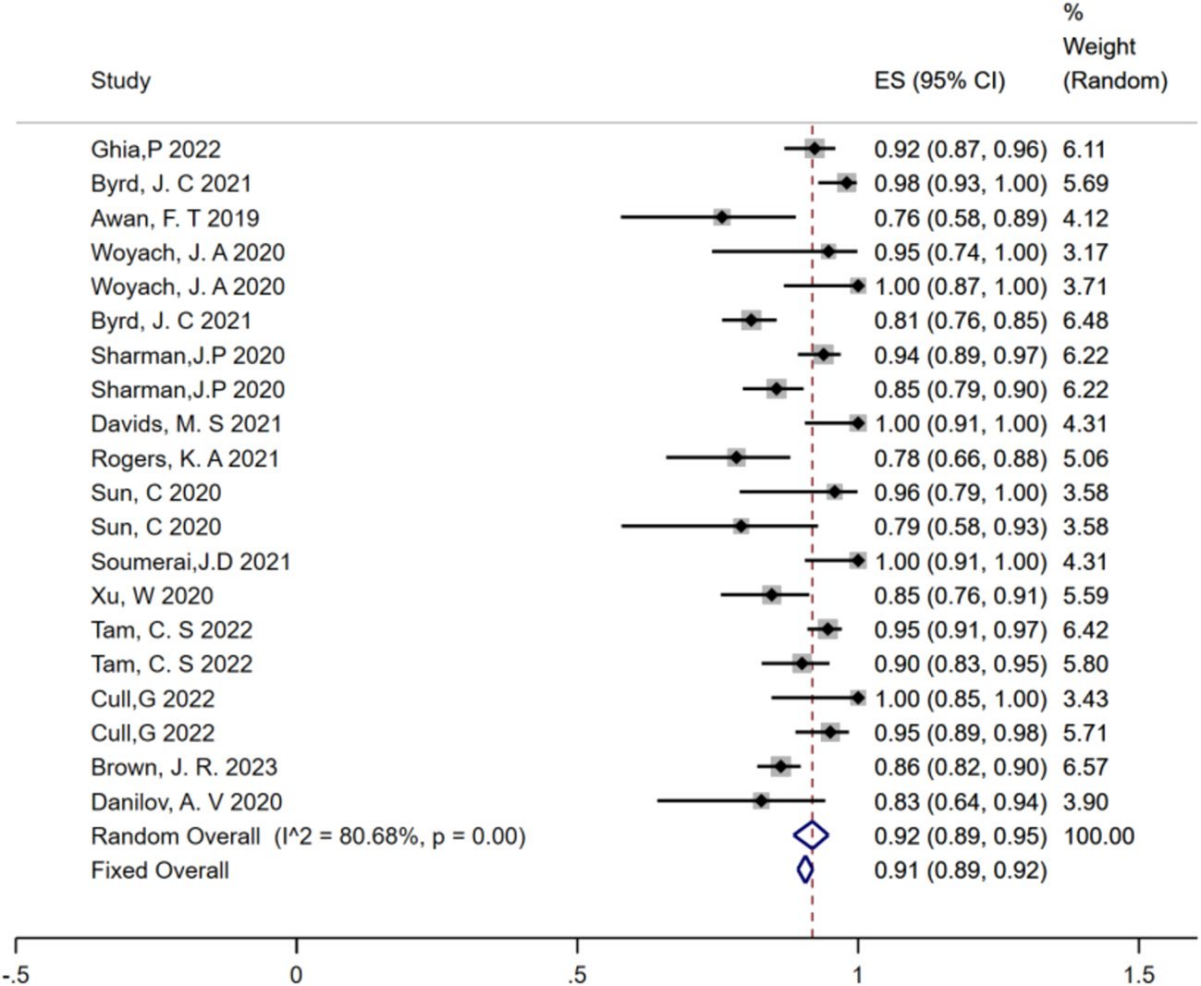
Pooled overall response rate (random effect model) of CLL

All 20 studies reported ORR and CR rates as clinical outcomes. The pooled ORR for new-generation BTKi was 92% (95% CI, 89–95%,* I*^2^ = 80.68%,* P* = 0.00), while the pooled CR rate was 10% (95% CI, 6–14%, *I*^2^ = 88.11%, *P* = 0.00) **[**Fig. 2**]**. Analysis using the random-effects model confirmed the considerable efficacy of new-generation BTKi treatment in CLL.

##### Sub-group analysis on age

All 20 studies evaluated the ORR and CR rates for the age group. The younger patients (< 65 years old) appeared to have higher ORR (95%, 95% CI, 88–99%) than older patients (≥ 65 years old) (90%, 95% CI, 86–93%). Both groups exhibited heterogeneity (*I*^2^ = 79.48%,* P* = 0.00 for the younger, and* I*^2^ = 80.44%, *P* = 0.00 for the older), so the random-effect model was used. The respective CR rate for the younger and older younger groups were 16% (95% CI, 5–30%, *I*^2^ = 90.03%, *P* = 0.00) and 6% (95% CI, 4–10%, *I*^2^ = 82.97%, *P* = 0.00). The pooled results showed that the younger group has better efficacy. **[**Supplementary Figure S2**]**.

##### Sub-group analysis on disease status

All 20 studies included the ORR and CR rates of BTKi therapy by disease status (TN-CLL vs RR-CLL). Statistical analysis of TN CLL patients showed that the pooled ORR was 96% (95% CI, 92–98%,* I*^2^ = 74.37%, *P* = 0.00), while the pooled CR rate was 16% (95% CI, 7–28%, *I*^2^ = 93.53%, *P* = 0.00). Five studies reported the ORR and CR rate of R/R CLL. The pooled ORR and CR rate were 90% (95% CI, 85–95%, *I*^2^ = 77.17%, *P* = 0.00) and 7% (95% CI, 4–10%, *I*^2^ = 56.68%, *P* = 0.04) respectively. The pooled outcomes indicated better efficacy in the TN-CLL group **[**Supplementary Figure S3**]**.

##### Sub-group analysis on treatment strategy

Sub-group analysis of new-generation BTKi combined with different treatment measures demonstrated that the pooled ORR and CR rate varied among different treatment strategies. BTKi monotherapy was given in sixteen studies, and the pooled ORR was 90% (95% CI, 88–91%,* I*^2^ = 80.88%, *P* = 0.00). Acalabrutinib monotherapy was reported in eight studies, and the pooled ORR was 87% (95% CI, 81–93%, *I*^2^ = 82.23%, *P* = 0.00). Seven studies used zanubrutinib monotherapy for CLL patients, and the pooled ORR was 93% (95% CI, 89–97%, *I*^2^ = 79.48%, *P* = 0.00). Pooled CR rate in BTKi monotherapy was 7% (95% CI, 4–12%, *I*^2^ = 85.85%, *P* = 0.00), which was 3% (95% CI, 1–6%, *I*^2^ = 61.78%, *P* = 0.00) and 13% (95% CI, 6–22%, *I*^2^ = 90.36%, *P* = 0.00) in acalabrutinib and zanubrutinib sub-group respectively. Only one study evaluated tirabrutinib, and the pooled ORR was 83% (95% CI, 64–94%), while the pooled CR rate was 7% (95% CI, 1–23%).

Moreover, four studies with 261 patients assessed BTKi combination (acalabrutinib combination) therapy, and the pooled ORR was 97% (95% CI, 94–99%, *I*^2^ = 46.41%, *P* = 0.13), while the pooled CR rate was 22% (95% CI, 8–42%, *I*^2^ = 86.09%, *P* = 0.00). Twelve studies reported the ORR and CR rate of acalabrutinib-based regimens. The pooled ORR and CR rate were 91% (95% CI, 86–95%, *I*^2^ = 83.18%, *P* = 0.00) and 8% (95% CI, 4–14%, *I*^2^ = 87.81%, *P* = 0.00) respectively **[**Supplementary Figure S4, S5, and S6**]**.

#### Survival

Several studies reported survival data for the 24-month OS rate and 24-month PFS rate. Pooled 24-month OS rate for CLL patients treated with BTKi was 94% (95% CI, 92–97%, *I*^2^ = 51.32%, *P* = 0.06). Sub-group analysis for the acalabrutinib monotherapy and zanubrutinib monotherapy showed a pooled 24-month OS rate of 92% (95% CI, 89–96%, *I*^2^ = 0.00%) and 95% (95% CI, 92–96%, *I*^2^ = 0.00%, *P* = 0.72), respectively **[**Supplementary Figure S7**]**. Pooled 24-month PFS rate for CLL patients treated with BTKi was 86% (95% CI, 82–90%, *I*^2^ = 72.16%, *P* = 0.00).

Sub-group analysis for the acalabrutinib monotherapy and zanubrutinib monotherapy showed a pooled 24-month PFS rate of 83% (95% CI, 75–90%, *I*^2^ = 57.74%, *P* = 0.05) and 86% (95% CI, 80–91%, *I*^2^ = 77.84%, *P* = 0.00), respectively **[**Supplementary Figure S8**]**. Sub-group analysis for the BTKi combination therapy and BTKi monotherapy showed a pooled 24-month OS rate of 96% (95% CI, 93–99%, *I*^2^ = 0.00%) and 93% (95% CI, 90–96%, I^2^ = 61.88%, *P* = 0.03), respectively. The pooled 24-month PFS rate for BTKi combination therapy was 94% (95% CI, 90–97%,* I*^2^ = 0.00%), while for BTKi monotherapy was 85% (95% CI, 80–89%, *I*^2^ = 65.28%, *P* = 0.00) **[**Supplementary Figure S9**].** When comparing survival according to disease status, TN patients had a higher pooled 24-month OS rate (95% vs. 82%), and 24-month PFS rate (89% vs. 77%) compared to R/R patients **[**Supplementary Figure S10**]**.

#### Immunoglobulin heavy-chain variable gene (IGHV) status

Four studies reported the ORR of IGHV status. The fixed-effects model meta-analysis (*I*^2^ = 28.1%, *P* = 0.249) demonstrated that there was no statistically significant difference in ORR between unmutated IGHV and mutated IGHV (RR = 1.10, 95%CI, 0.99–1.21, *P* = 0.07) [Supplementary Figure S11].

### Toxicity

AEs were reported in all studies; neutropenia, anemia, and thrombocytopenia are the main hematological AEs. The pooled rate of grade ≥ 3 neutropenia was 17% (95% CI, 13–22%, *I*^2^ = 83.49%, *P* = 0.00), grade ≥ 3 anemia was 4% (95% CI, 2–7%, *I*^2^ = 83.01%, *P* = 0.00), and grade ≥ 3 thrombocytopenia was 5% (95% CI, 3–8%, *I*^2^ = 79.10%, *P* = 0.00). Severe non-hematological AEs mainly included diarrhea, fatigue, upper respiratory tract infection, atrial fibrillation, and hypertension. The pooled rate of grade ≥ 3 diarrhea was 1% (95% CI, 1–2%, *I*^2^ = 24.68%, *P* = 0.18), while the pooled rate of grade ≥ 3 fatigue was 1% (95% CI, 1–2%, *I*^2^ = 0.00%, *P* = 0.93). The pooled rate of grade ≥ 3 upper respiratory tract infection was 1% (95% CI, 0–2%, *I*^2^ = 51.00%, *P* = 0.01). The pooled rate of grade ≥ 3 atrial fibrillation and hypertension was 1% (95% CI, 1–2%, *I*^2^ = 43.06%, *P* = 0.03) and 4% (95% CI, 2–7%, *I*^2^ = 76.57%, *P* = 0.00), respectively **[**Supplementary Figure S12; Table 3]. Table 4 illustrated the pooled rates of grade ≥ 3 AEs in both BTKi monotherapy and BTKi combination therapy. The pooled rates of grade ≥ 3 upper respiratory tract infection and atrial fibrillation were both 1%. BTKi monotherapy exhibited a higher pooled rate of grade ≥ 3 hypertension (5% vs. 2%) compared to BTKi combination therapy. Conversely, the pooled rates of other grade ≥ 3 AEs were consistently lower in BTKi monotherapy when compared to BTKi combination therapy. Table 5 demonstrated the pooled results of grade ≥ 3 AEs between acalabrutinib and zanubrutinib monotherapy. The pooled rates of grade ≥ 3 neutropenia, anemia, and thrombocytopenia in acalabrutinib monotherapy were 14%, 7%, and 5% respectively. The pooled rates of grade ≥ 3 neutropenia, anemia, and thrombocytopenia in zanubrutinib monotherapy were 19%, 2%, and 4% respectively. Zanubrutinib monotherapy had a similar pooled rate of grade ≥ 3 upper respiratory tract infection (2% vs. 1%), and grade ≥ 3 hypertension (6% vs. 4%) compared to acalabrutinib monotherapy. The pooled rates of other grade ≥ 3 AEs were both 1%.

**Table 3:** Summary of the safety meta-analysis

Adverse event	≥ Grade 3				
	Effect size, % (95% CI)	*I*^2^ value (%)	*P* value (%)	*P* value (Begg’s)	*P* value (Egger’s)
Neutropenia	17 (13–22)	83.49	0.00	0.773	0.677
Thrombocytopenia	5 (3–8)	79.10	0.00	0.192	0.225
Anemia	4 (2–7)	83.01	0.00	1.000	0.841
Diarrhea	1 (1–2)	24.68	0.18	0.558	0.990
Fatigue	1 (1–2)	0.00	0.93	0.692	0.239
Upper respiratory tract infection	1 (0–2)	51.00	0.01	0.843	0.929
Atrial fibrillation	1 (1–2)	43.06	0.03	0.537	0.476
Hypertension	4 (2–7)	76.57	0.00	0.753	0.102

**Table 4:** Pooled rates of grade ≥ 3 AEs between BTKi monotherapy and BTKi combination therapy

Adverse event	≥ Grade 3	
	Monotherapy	Combination therapy
Neutropenia	16% (13–20%)	22% (4–48%)
Thrombocytopenia	4% (2–6%)	11% (7–15%)
Anemia	4% (2–7%)	5% (3–8%)
Diarrhea	1% (0–2%)	3% (1–6%)
Fatigue	1% (1–2%)	2% (0–4%)
Upper respiratory tract infection	1% (0–2%)	1% (0–3%)
Atrial fibrillation	1% (0–2%)	1% (0–3%)
Hypertension	5% (3–7%)	2% (0–6%)

**Table 5:** Pooled results of grade ≥ 3 AEs between acalabrutinib and zanubrutinib monotherapy

Adverse event	≥ Grade 3	
	Acalabrutinib	Zanubrutinib
Neutropenia	14% (11–19%)	19% (12–27%)
Thrombocytopenia	5% (2–8%)	4% (1–8%)
Anemia	7% (4–11%)	2% (0–5%)
Diarrhea	1% (0–2%)	1% (0–2%)
Fatigue	1% (1–2%)	1% (0–2%)
Upper respiratory tract infection	1% (0–2%)	2% (0–6%)
Atrial fibrillation	1% (0–3%)	1% (0–2%)
Hypertension	4% (2–6%)	6% (2–10%)

### Analysis of publication bias

**Fig. 3:**
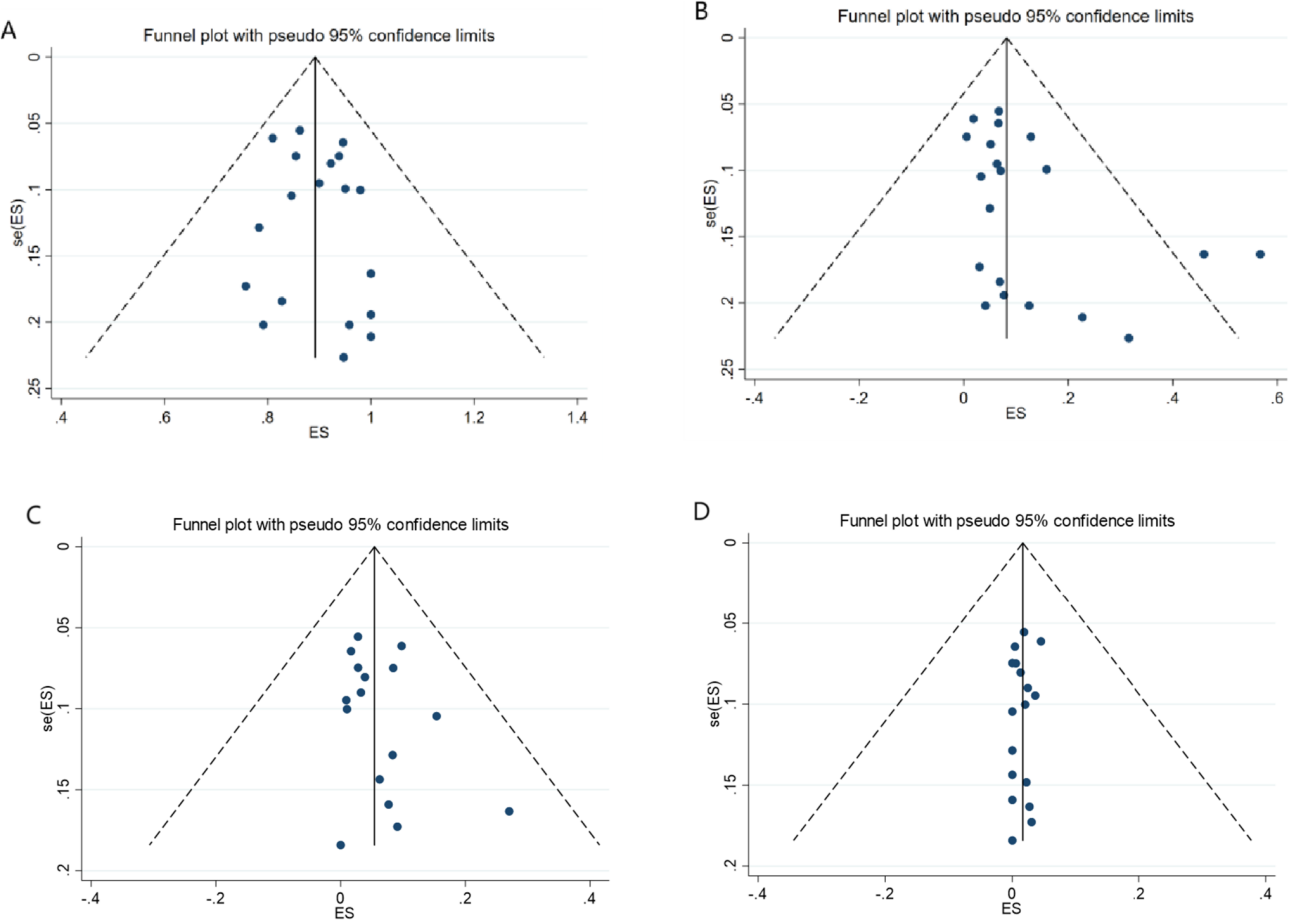
**A** The funnel plot of the total overall response rate; **B** The funnel plot of the total complete remission rate; **C** The funnel plot of total grade ≥ 3 thrombocytopenia rate; **D** The funnel plot of total grade ≥ 3 atrial fibrillation rate

In this study, Egger’s and Begg’s tests were conducted on ORR and CR rate to determine publication bias Table 6. It was regarded as no publication bias if the *P* value > 0.05 was met in both methods. The pooled ORR assessment results did not show significant publication bias among included studies. For CR rate, publication bias occurred in the total cohort, TN-CLL, and acalabrutinib-based groups. No publication bias was found in the Egger’s and Begg’s tests for AEs (grade ≥ 3) regarding safety outcomes. The funnel chart of Egger’s and Begg’s is shown partly included in Fig. 3.

**Table 6:** Summary of the ORR and CRR meta-analysis

Parameters	Effect size, % (95% CI)	*I*^2^ value (%)	*P* value	*P* value (Begg’s)	*P* value (Egger’s)
ORR	92% (89–95%)	80.68	0.00	0.845	0.389
CRR	10% (6–14%)	88.11	0.00	0.011	0.031
Age-ORR					
Age ≥ 65	90% (86–93%)	80.44	0.00	0.815	0.697
Age < 65	95% (88–99%)	79.48	0.00	0.249	0.983
Age-CRR					
Age ≥ 65	6% (4–10%)	84.53	0.00	0.482	0.223
Age < 65	16% (5–30%)	90.03	0.00	0.343	0.336
Disease state-ORR					
TN-CLL	96% (92–98%)	74.37	0.00	0.753	0.214
RR-CLL	90% (85–95%)	77.17	0.00	1.000	0.664
Disease state-CRR					
TN-CLL	16% (7–28%)	93.53	0.00	0.045	0.020
RR-CLL	7% (4–10%)	56.68	0.04	1.000	0.879
Therapy-ORR					
Monotherapy	90% (88–91%)	80.88	0.00	0.787	0.716
Combination therapy	97% (94–99%)	46.41	0.13	1.000	0.259
Therapy-CRR					
Monotherapy	7% (4–12%)	85.85	0.00	0.149	0.105
Combination therapy	22% (8–42%)	86.09	0.00	0.734	0.459
Monotherapy-ORR					
Acalabrutinib	87% (81–93%)	82.23	0.00	0.618	0.955
Zanubrutinib	93% (89–97%)	79.48	0.00	0.764	0.263
Monotherapy-CRR					
Acalabrutinib	3% (1–6%)	61.78	0.01	0.319	0.128
Zanubrutinib	13% (6–22%)	90.36	0.00	0.133	0.104
Intervention-ORR					
Acalabrutinib-based	91% (86–95%)	83.18	0.00	1.000	0.513
Intervention-CRR					
Acalabrutinib-based	8% (4–14%)	87.81	0.00	0.046	0.114

### Sensitivity analysis

**Fig. 4:**
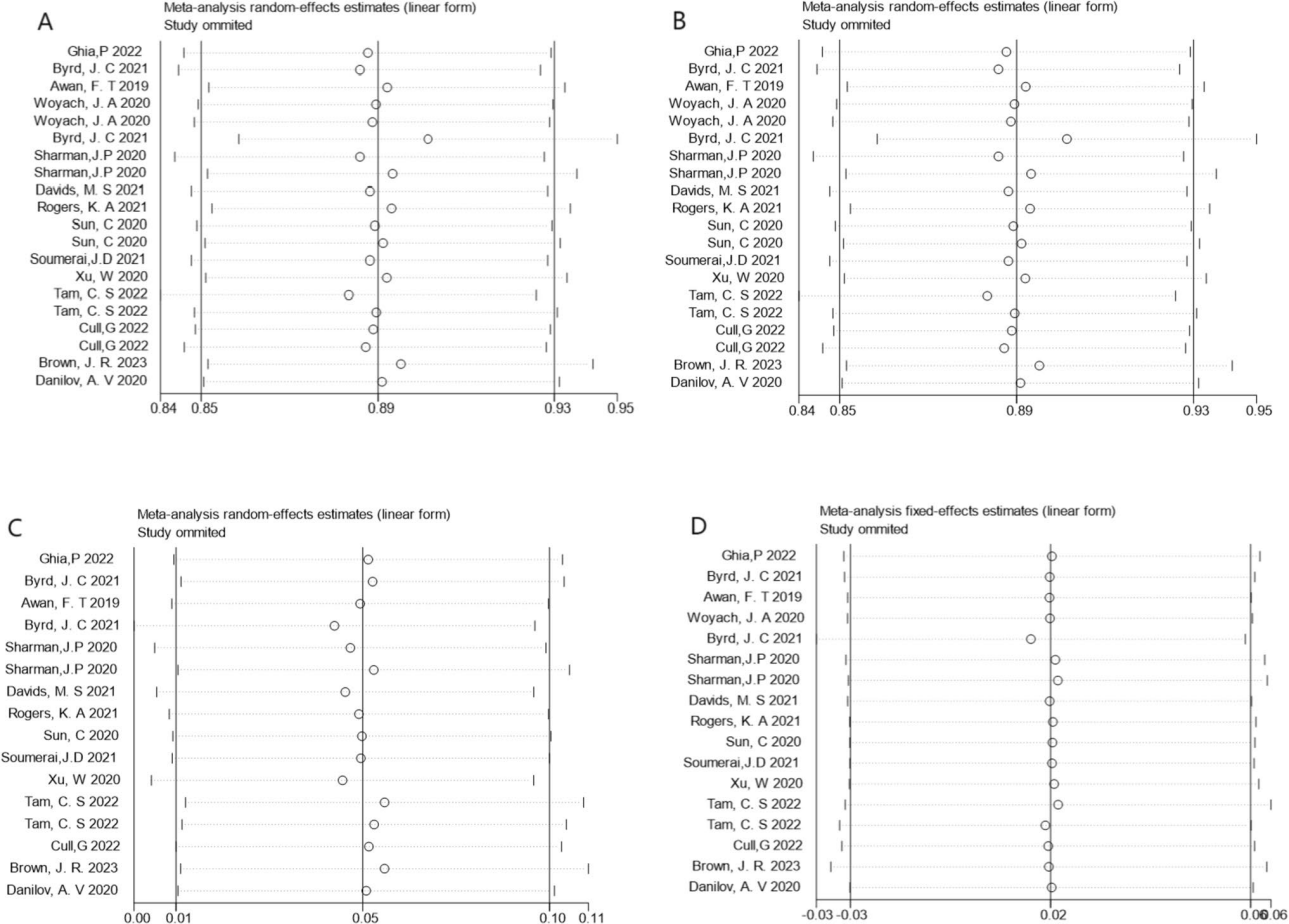
The graph of sensitivity analysis. **A** The sensitivity analysis of the total overall response rate; **B** the sensitivity analysis of the total complete response rate; **C** the sensitivity analysis of total grade ≥ 3 thrombocytopenia rate; **D** the sensitivity analysis of total grade ≥ 3 atrial fibrillation rate

Sensitivity analysis was conducted by removing individual studies from highly heterogeneous aggregated results one by one. When omitting the study, the pooled analysis of ORR, CR rate, and grade ≥ 3 AE did not show significant changes, indicating that our comprehensive results are reliable. Some of the results are shown in Fig. 4.

## Discussion

Chronic lymphocytic leukocytosis (CLL) is a B-cell malignant tumor characterized by the clonal aggregation of CD5 + and CD19 + B cells in the bone marrow and peripheral blood. The World Health Organization (WHO) classifies CLL as an indolent B-cell lymphoma ^[27]^. The prognosis of patients with CLL is very heterogeneous, some patients have an inert course of disease and do not need treatment for life, while others invade the course of disease, showing early treatment indications. Until recently, chemoimmunotherapy with fludarabine, rituximab, and cyclophosphamide, rituximab and bendamustine, or chlorambucil and obinutuzumab was the standard care for TN patients who were physically fit (fludarabine, rituximab, and cyclophosphamide) or had coexisting conditions (chlorambucil plus bendamustine/obinutuzumab and rituximab). The only way to cure CLL is allogeneic hematopoietic stem cell transplantation, but unfortunately, most patients are not suitable for transplantation and can only manage the disease and symptoms with drugs.

The BCR signaling pathway is involved in the pathogenesis of CLL. There is no doubt that the BCR signaling pathway is essential for maintaining the survival, proliferation, and development of CLL cells. Drugs inhibiting the enzymes involved in the BCR pathway, specifically BTK, are the standard care for treating CLL nowadays. Ibrutinib is the world’s first covalent, irreversible BTKi approved by the US FDA. RESONATE^[28]^, RESONATE-2^[29]^, and ECOG 1912^[30]^ respectively established the efficacy of ibrutinib in R/R CLL, TN CLL ≥ 65 years old, and TN CLL ≤ 70 years old. A recent pooled analysis of four clinical trials showed that in TN CLL with TP53 aberrations, the 4-year PFS rate was 79% and the 4-year total survival (OS) rate was 88%^[31]^. Therefore, ibrutinib is recommended as the preferred treatment for CLL patients with TP53 aberrations. With the prolongation of survival brought by the continuous optimization of treatment, the proportion of elderly patients with CLL increases, while elderly patients are often complicated with cardiovascular and cerebrovascular diseases, hypertension, diabetes, and so on. In this context, clinical attention to the safety of treatment is increasingly important. The safety results of the 8-year follow-up of RESONATE-2 showed that the incidence of hypertension was more than 20% and the incidence of atrial fibrillation was about 10% in the last 3 years^[32]^. Additionally, the FDA updated the manual of ibrutinib to warn of cardiac safety issues in May 2022. Fatal and serious cardiac failure and cardiac arrhythmias have occurred after ibrutinib administration. Among 4896 patients who underwent clinical trials of ibrutinib (including monotherapy or combination therapy), 1% of patients died from cardiac causes or sudden death. These AEs occurred in patients both with and without preexisting hypertension or cardiac comorbidities. Patients with cardiac comorbidities may have a greater risk^[33]^. Considering the toxicity of ibrutinib, it has been moved from “Preferred regimens” to “Other recommended regimens” in the National Comprehensive Cancer Network Guidelines (NCCN Guidelines Version 1.2023) for CLL. Patients’ cardiovascular function must be strictly evaluated before using ibrutinib^[34]^.

Acalabrutinib, zanubrutinib, and tirabrutinib are new covalent BTKis that exhibit greater selectivity for BTK compared to ibrutinib and were initially anticipated to have a more favorable safety profile. In the phase II clinical trial, 33 patients with CLL who were intolerant to ibrutinib were treated with acalabrutinib, 72% had no recurrent adverse reactions related to ibrutinib, and 13% had adverse reactions related to ibrutinib, but the degree was reduced. With a median follow-up of 19 months, ORR was 76% including 1 patient who achieved CR^[14]^. SEQUOIA ^[23]^study evaluated the efficacy and safety of zanubrutinib versus bendamustine plus rituximab (BR) in the first-line treatment of elderly or young CLL patients with comorbidities without del (17p). At a median follow-up of 26.2 months, the 24-month PFS rate assessed by IRC was 85.5% in the zanubrutinib group and 69.5% in the BR group. PFS was significantly improved in the zanubrutinib group compared with the BR group (HR = 0.42, two-sided *P* < 0.0001). Subgroup analysis showed that regardless of age, gender, high-risk disease status, and other key stratification, PFS in zanubrutinib group were superior to the BR group. In the ELEVATE R/R study^[16]^, either acalabrutinib or ibrutinib was randomly assigned to 533 patients who were previously treated high-risk CLL—del (17p) or del (11q). The IRC-assessed ORR was 81.0% (95% CI, 75.8–85.2) for acalabrutinib and 77.0% (95% CI, 71.5–81.6). The median PFS of acalabrutinib (38.4 months in both groups) was non-inferior to ibrutinib. However, compared with ibrutinib, the incidence of atrial fibrillation/flutter (9.4% vs 16%; *P* = 0.02), hypertension (9.4% vs 23.2%), and bleeding events (38% vs 51.3%) were lower. There are differences in the discontinuation rates caused by AE, with acalabrutinib being 14.7% and ibrutinib being 21.3%. Zanubrutinib or ibrutinib was randomly assigned to 652 patients who had previously received CLL treatment in the ALPINE study^[25]^. Compared with ibrutinib, zanubrutinib treatment can improve the overall response (86.2% vs 75.7%, *P* < 0.01) and the 24-month PFS incidence (78.4% vs 65.9%, *P* = 0.002). Zanubrutinib was associated with a lower cumulative incidence of atrial fibrillation/flutter (5.2% vs 13.3%), but the incidence rate of neutropenia increased (29.3% vs 24.4%), while the infection rate did not increase (71.3% vs 73.1%). Compared to ibrutinib, events leading to discontinuation of medication with zanubrutinib are less common (14.5% vs 22.2%). Based on the results of these studies, zanubrutinib and acalabrutinib are preferred over ibrutinib due to their favorable safety profile, and zanubrutinib has superior efficacy compared with ibrutinib.

Our meta-analysis showed that the pooled ORR and CR rate of new-generation BTKi-based treatment for CLL were 92% and 10%, respectively, confirming their good efficacy. However, the* I*^2^ values for ORR and CR rate were 80.68% and 88.73%, which was quite heterogeneous. Meanwhile, the reason why the heterogeneity of subgroup analysis results did not decrease may be due to significant differences in sample size and individual heterogeneity in each study. Among all studies, six had a sample size of less than 30, while one study involved 327 patients.

According to the results of the analysis, the following new-generation BTKi-based therapy conditions yielded higher efficacy: < 65 years old, TN-CLL, and BTKi combination therapy. Sub-group analysis showed that the ORR and CR rates from acalabrutinib monotherapy for CLL were 87% and 3%, respectively, while zanubrutinib monotherapy showed OR and CR rates of 93% and 13%, respectively. The ORR and CR rates of acalabrutinib combined with chemotherapy were 97% and 22%. These results indicated that adding acalabrutinib to chemotherapy may improve efficacy. The ORR and CR rates of BTKi combination therapy were higher than those of BTKi monotherapy, suggesting that the efficacy of BTKi combination therapy was superior to BTKi monotherapy. Furthermore, zanubrutinib monotherapy yielded higher efficacy than acalabrutinib monotherapy, indicating that zanubrutinib may be the first choice in monotherapy for CLL compared to acalabrutinib. The head-to-head RCTs are still needed to compare the efficacy between zanubrutinib monotherapy and acalabrutinib monotherapy. Furthermore, we explored the impact of IGHV status on the efficacy of new-generation BTKi treatment. The results showed that there was no statistically significant difference in ORR between unmutated IGHV and mutated IGHV.

Ibrutinib had shown impressive survival data in CLL. In this study, the 24-month OS and PFS rates of patients who received the new-generation BTKi-based regimen were 94% and 86%, respectively. According to the subgroup analysis, the rates of BTKi combination therapy were higher than BTKi monotherapy (24-month OS: 96% vs 93%; 24-month PFS: 94% vs 85%). Zanubrutinib monotherapy was slightly better than acalabrutinib monotherapy (24-month OS: 95% vs 92%; 24-month PFS: 86% vs 83%). However, most studies did not reach the median OS, and pooling long-term survival outcomes was impossible due to the limited follow-up duration. The effects of new-generation BTKi on survival need to be further evaluated by extending the follow-up time. In our meta-analysis of ≥ grade 3 AEs, we found that hematology AEs were the most common AEs, with an incidence of 5–14%. The main manifestations as cytopenia, including neutropenia, anemia, and thrombocytopenia. The highest incidence of non-hematological AEs was hypertension, which was 4%. Other non-hematological AEs including diarrhea, fatigue, and upper respiratory tract infection were all 1%. Previous studies had shown that ibrutinib caused a high incidence of atrial fibrillation. This study’s pooled grade ≥ 3 atrial fibrillation rate was only 1%. The results of subgroup analysis indicated that BTKi combination therapy was associated with higher or similar rates of most AEs compared to BTKi monotherapy, which to some extent limited the utilization of BTKi combination therapy. The use of acalabrutinib monotherapy was associated with lower rates of neutropenia and hypertension and higher rates of thrombocytopenia and anemia compared to zanubrutinib monotherapy. No numerical differences in grade ≥ 3 diarrhea, fatigue, or upper respiratory tract infection were found between both acalabrutinib and zanubrutinib monotherapy. Differences in the safety profile between acalabrutinib and zanubrutinib can assist clinicians in selecting a specific BTKi based on patients’ comorbidities and/or preferences.

This meta-analysis has several limitations. (1) Our analysis has certain limitations due to the nature of the included studies, which are mostly prospective phase I/II clinical trials. The single-arm trials make difficult robust comparisons with other treatment options. (2) In our analysis, we identified significant heterogeneity, which could be attributed to the small sample size in some included studies and the infrequent incidence of certain events. (3) We included studies on acalabrutinib and zanubrutinib which accounted for the majority of the interventions, while tirabrutinib was only included in one. No research on orelabrutinib has been retrieved. The efficacy and safety of tirabrutinib and orelabrutinib cannot be well evaluated. (4) The survival data of most studies are incomplete. Most studies did not reach the median OS and PFS. Therefore, we only analyzed the 24-month OS and PFS from several studies. (6) Among all the retrieved studies, only acalabrutinib was used in combination therapy, while zanubrutinib and tirabrutinib were both used as monotherapy. (7) Since most studies have not separately shown the efficacy of gene mutations in patients with CLL, gene-related subgroup analysis has not been all carried out.

## Conclusions

Overall, this meta-analysis has confirmed the excellent efficacy and safety of new-generation BTKi for CLL. The efficacy of BTKi combination therapy is superior to BTKi monotherapy, but its incidence of AEs is higher than monotherapy. The increased occurrence of adverse effects is attributed to the combination of multiple drugs, which raises the risk of drug interactions and side effects. Therefore, exploring safer combination treatment strategies is expected to become one of the future research priorities. Among the BTKi monotherapy, we mainly compare acalabrutinib and zanubrutinib. Zanubrutinib may be the preferred choice in monotherapy for CLL compared to acalabrutinib; both acalabrutinib and zanubrutinib have their advantages and disadvantages in terms of AEs, but the incidence of atrial fibrillation is low for both. Toxicity should be monitored by clinicians, and timely prevention and intervention should be provided as well. To verify our findings and establish the impact of new-generation BTKi on CLL, it is crucial to conduct large-scale multicenter studies and RCTs. Additionally, further studies are needed to determine the optimal schedule of BTKi for CLL treatment.

The following supporting information can be downloaded at: https://doi.org/10.5281/zenodo.7970370, Table S1: Search algorithm; Figure S1: Quality assessment of included randomized studies; Figure S2: Forest plots assessing the effect of age (≥ 65 vs < 65) on (A) ORR; (B) CRR; Figure S3: Forest plots assessing the effect of disease status ( TN-CLL vs RR-CLL) on (A) ORR; (B) CRR; Figure S4:Forest plots assessing the effect of treatment strategy (BTKi monotherapy vs BTKi combination therapy) on (A) ORR; (B) CRR; Figure S5: Forest plots assessing the effect of treatment strategy (acalabrutinib monotherapy vs. zanubrutinib monotherapy) on (A) ORR; (B) CRR; Figure S6: Forest plots assessing the effect of treatment strategy (acalabrutinib-based regimen) on (A) ORR; (B) CRR; Figure S7: Forest plots assessing the 24-months OS (A) BTKi; (B) acalabrutinib monotherapy; (C) zanubrutinib monotherapy; Figure S8: Forest plots assessing the 24-months PFS (A) BTKi; (B) acalabrutinib monotherapy; (C) zanubrutinib monotherapy; Figure S9: Forest plots assessing the effect of BTKi combination therapy vs BTKi monotherapy (A) 24-months OS; (B) 24-months PFS; Figure S10: Forest plots assessing the effect of disease status (TN-CLL vs RR-CLL) (A) 24-months OS; (B) 24-months PFS; Figure S11: Forest plot of the ORR for treatment with the unmutated IGHV vs. mutated IGHV (fixed effect model). RR is the effect size; Figure S12: Forest plots for pooled grade ≥ 3 (A) neutropenia; (B) anemia; (C) thrombocytopenia; (D) diarrhea; (E) fatigue; (F) upper respiratory tract infection; (G) atrial fibrillation; (H) hypertension.

### Supplementary Information

Below is the link to the electronic supplementary material.

Supplementary file1 (DOCX 4326 KB)

Supplementary file2 (DOCX 21 KB)

## Data Availability

Data were extracted and analyzed from published articles available and accessible in the shared database. All datasets generated during the study are available upon reasonable request from the corresponding authors.
