# Supplementary material for: Efficacy and safety of new-generation Bruton tyrosine kinase inhibitors in chronic lymphocytic leukemia/small lymphocytic lymphoma: a systematic review and meta-analysis
Source: Ann Hematol. 2023 Oct 16;103(7):2231–44. doi: 10.1007/s00277-023-05486-x (PMC11224099; doi:10.1007/s00277-023-05486-x)
Supplement: Supplementary file 1 — Supplementary file1 (DOCX 4326 KB) [file 277_2023_5486_MOESM1_ESM.docx]

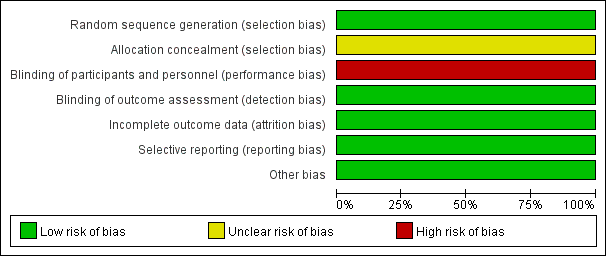


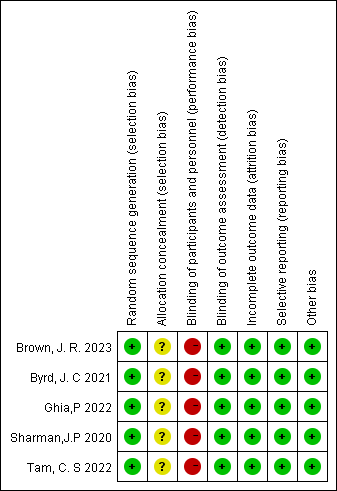


**Figure S1.** Quality assessment of included randomized studies

**Figure S2.** Forest plots assessing the effect of age ( ≥ 65 vs < 65) on (A) ORR; (B) CRR.


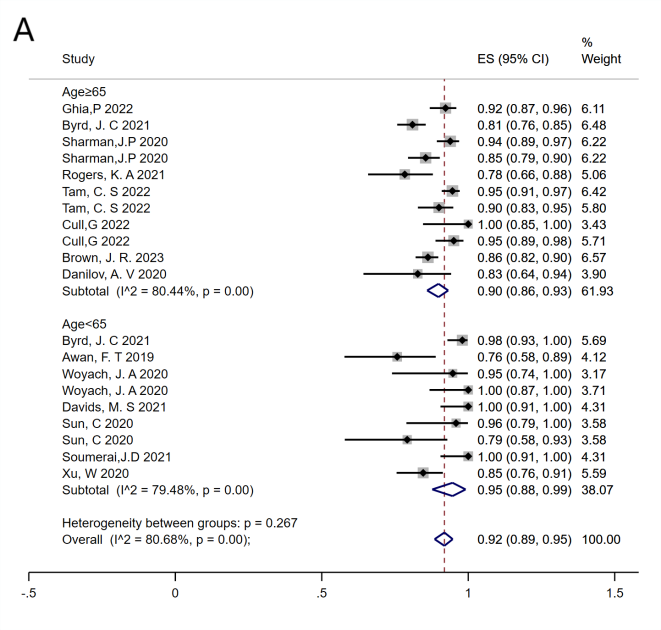

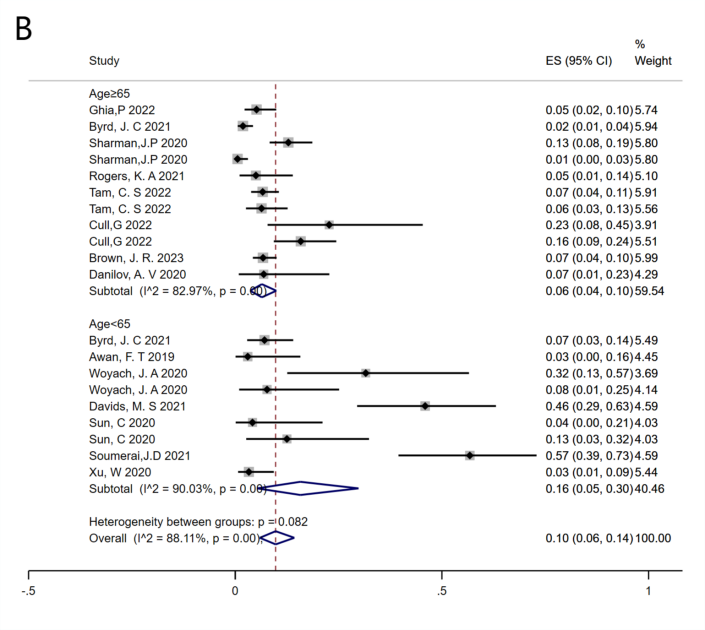

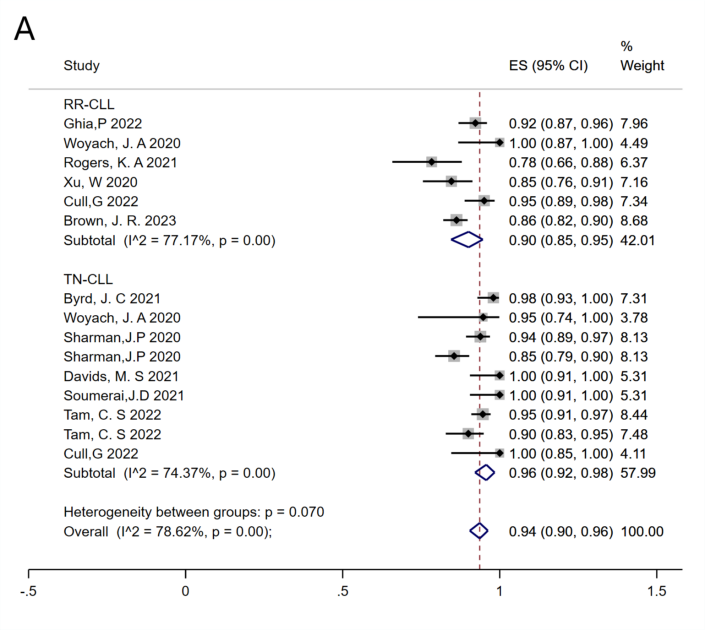

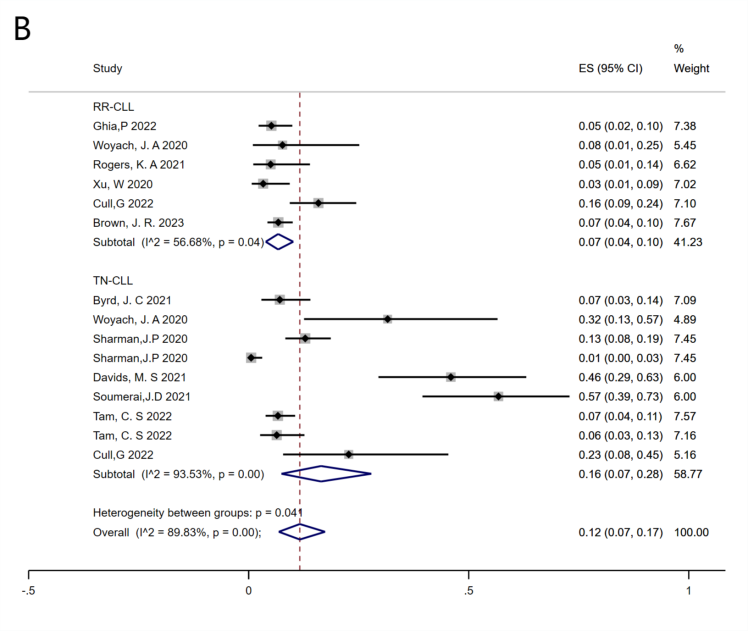


**Figure S3.** Forest plots assessing the effect of disease status ( TN-CLL vs RR-CLL) on (A) ORR; (B) CRR.


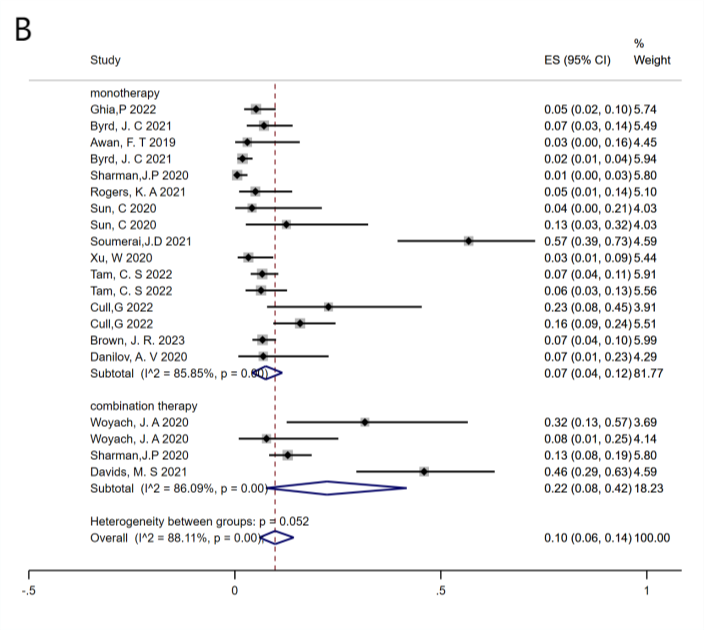

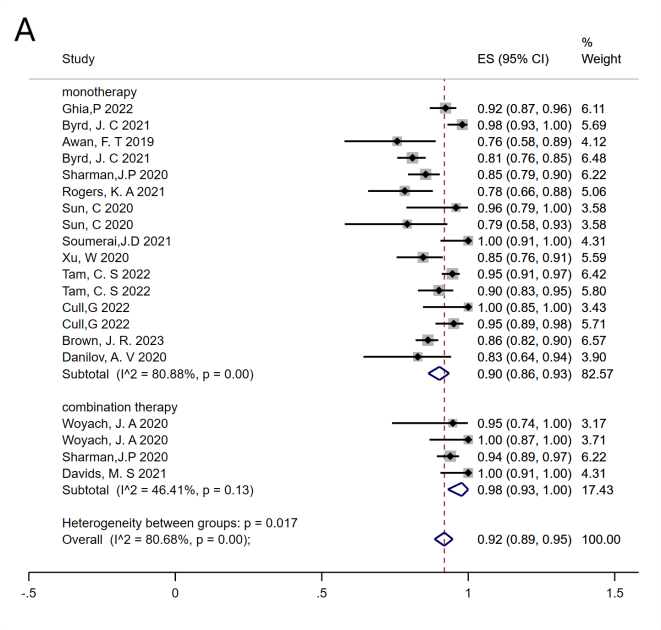


**Figure S4.** Forest plots assessing the effect of treatment strategy (BTKi monotherapy vs BTKi combination therapy) on (A) ORR; (B) CRR.


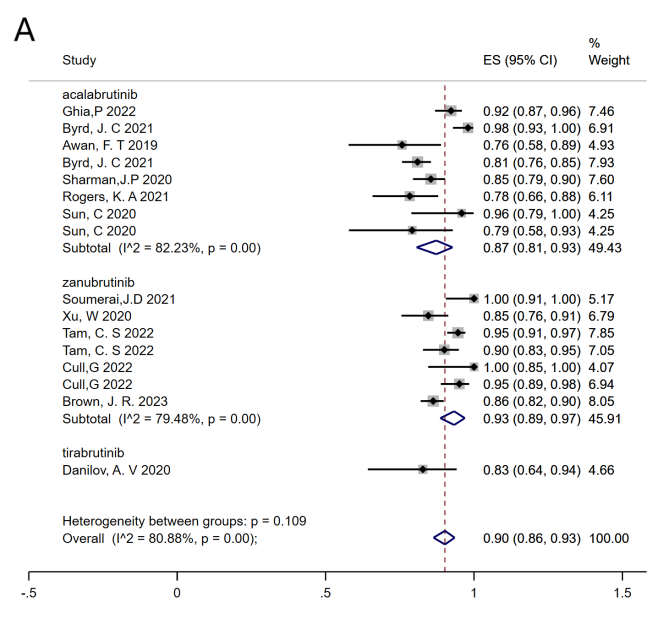

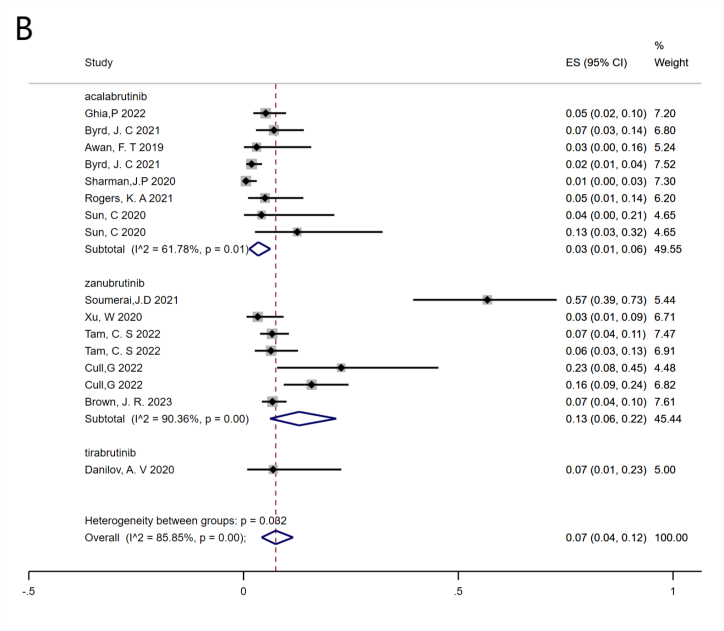


**Figure S5.** Forest plots assessing the effect of treatment strategy (acalabrutinib monotherapy vs. zanubrutinib monotherapy) on (A) ORR; (B) CRR.


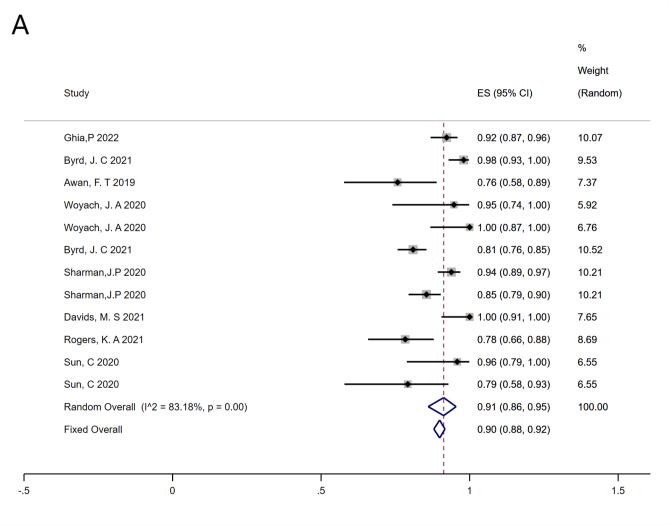

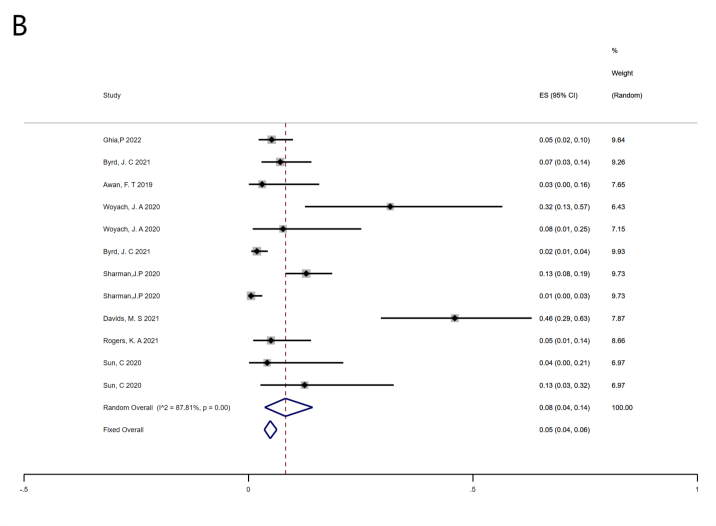


**Figure S6.** Forest plots assessing the effect of treatment strategy (acalabrutinib-based regimen) on (A) ORR; (B) CRR.


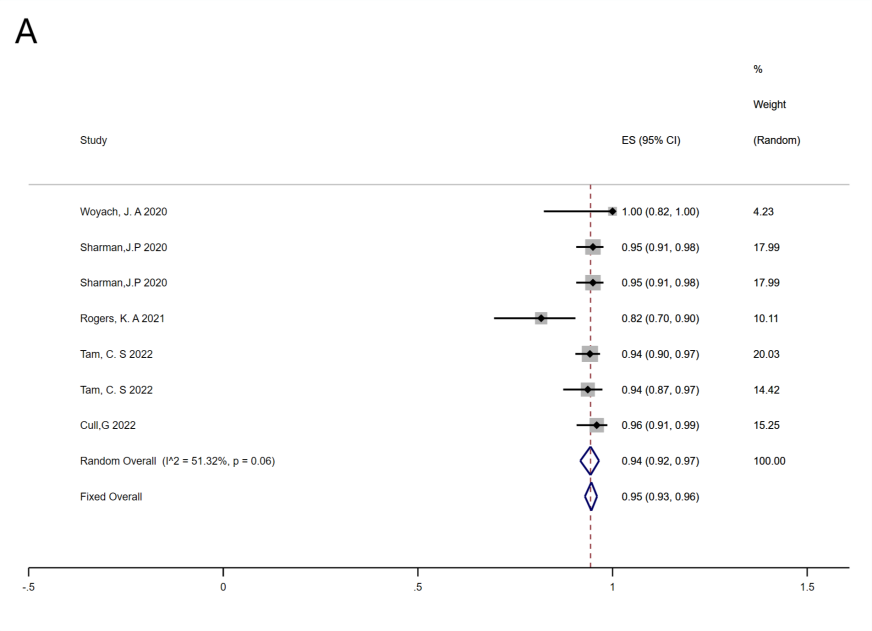


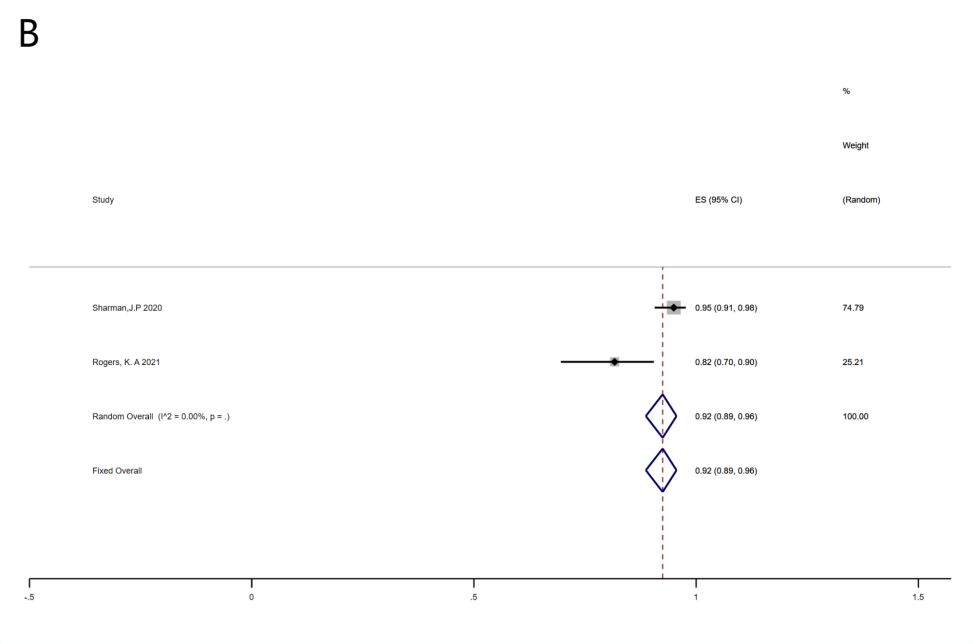


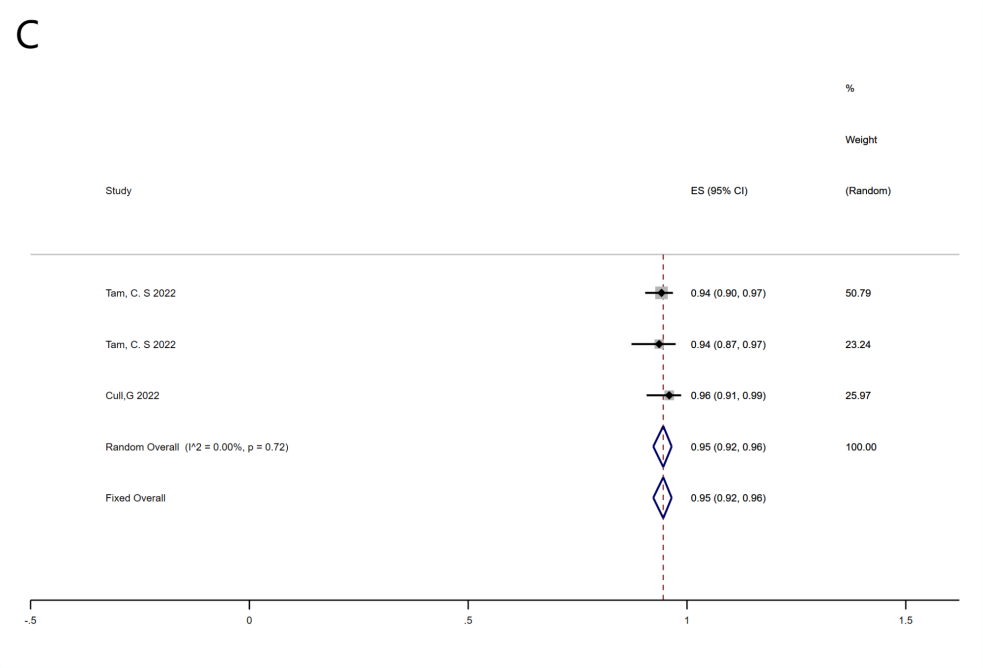


**Figure S7.** Forest plots assessing the 24-months OS (A) BTKi; (B) acalabrutinib monotherapy; (C) zanubrutinib monotherapy.


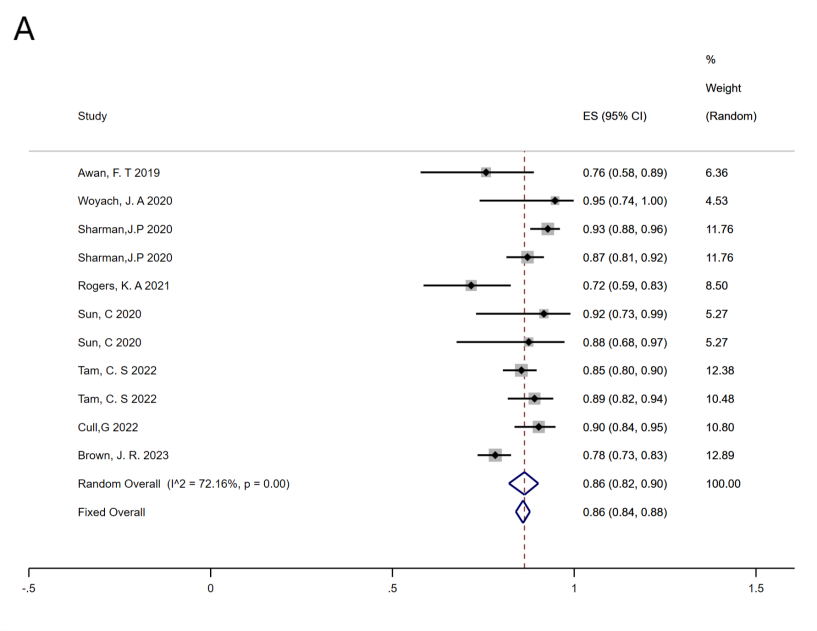


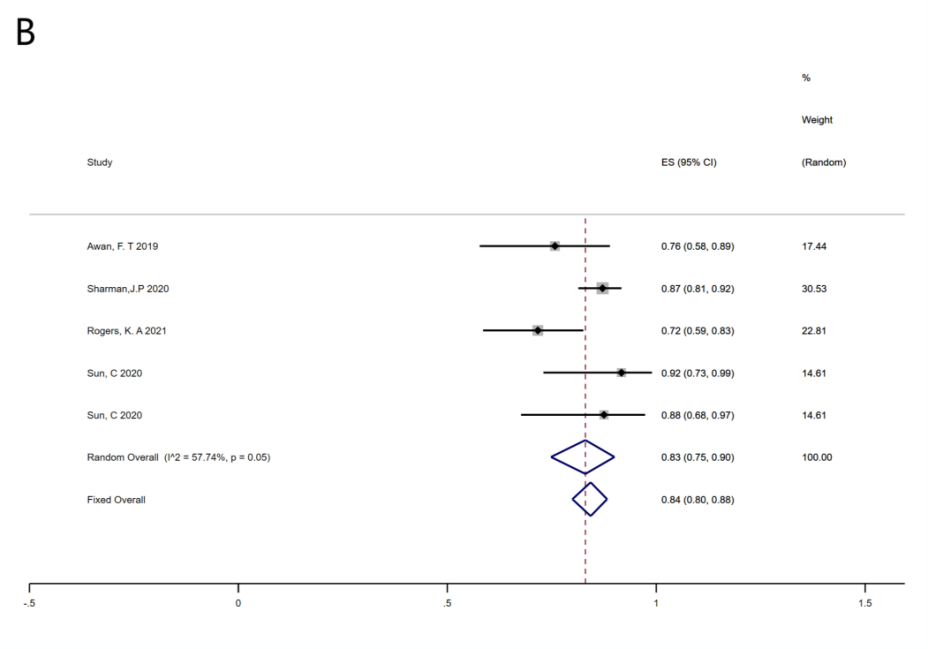


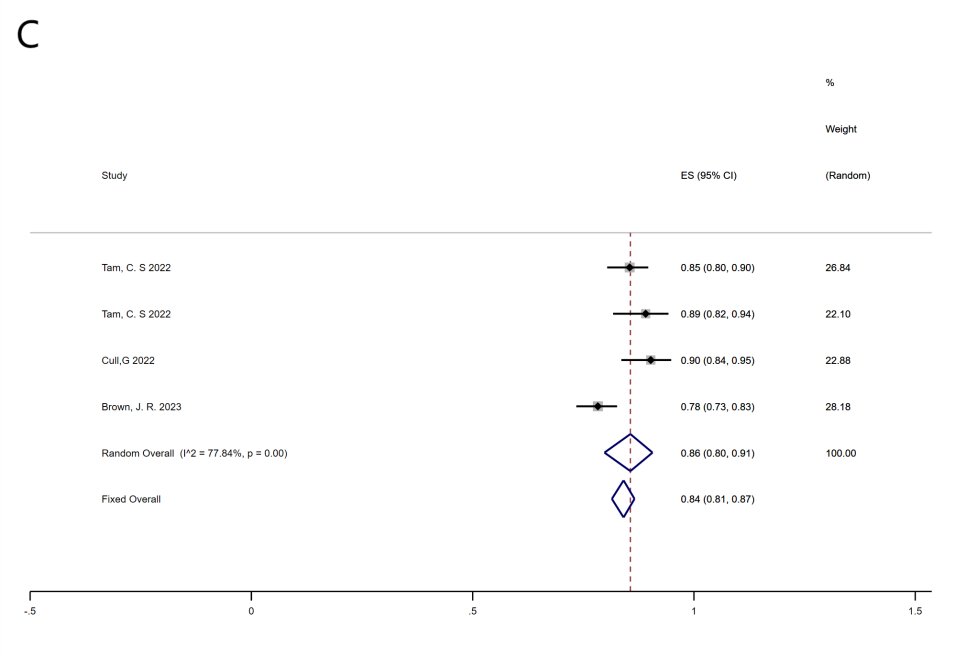


**Figure S8.** Forest plots assessing the 24-months PFS (A) BTKi; (B) acalabrutinib monotherapy; (C) zanubrutinib monotherapy.


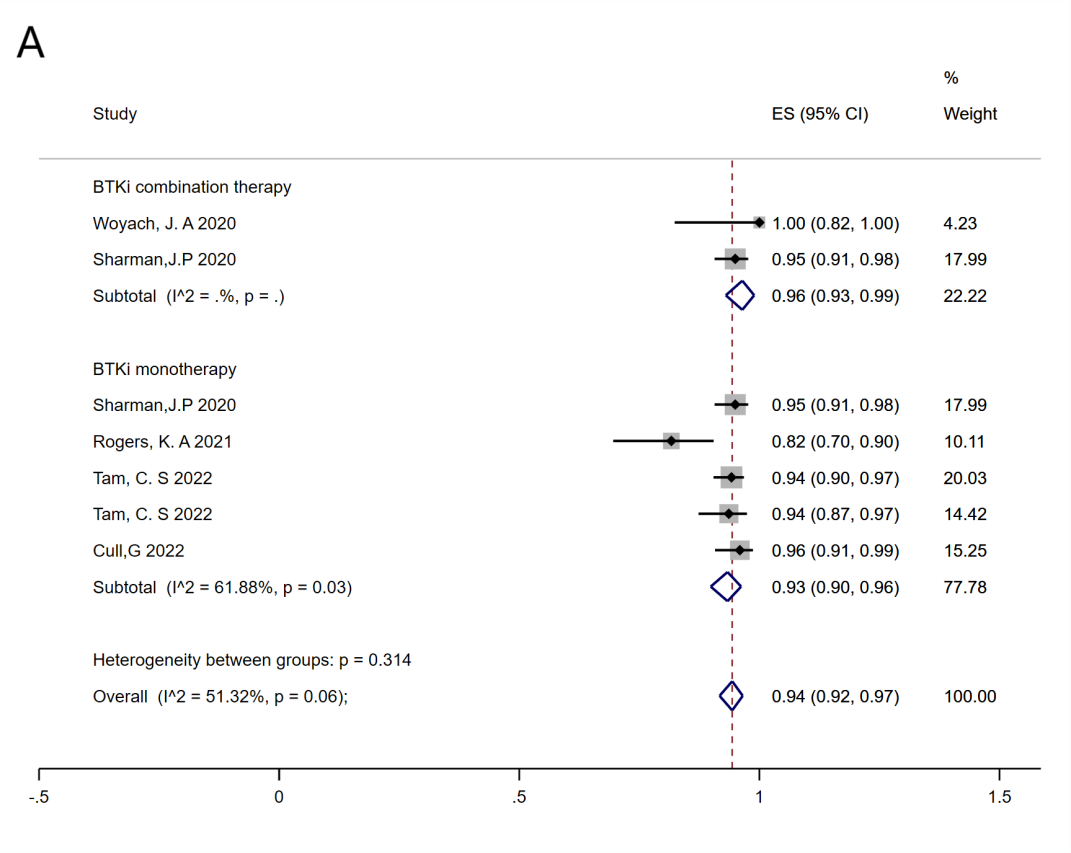


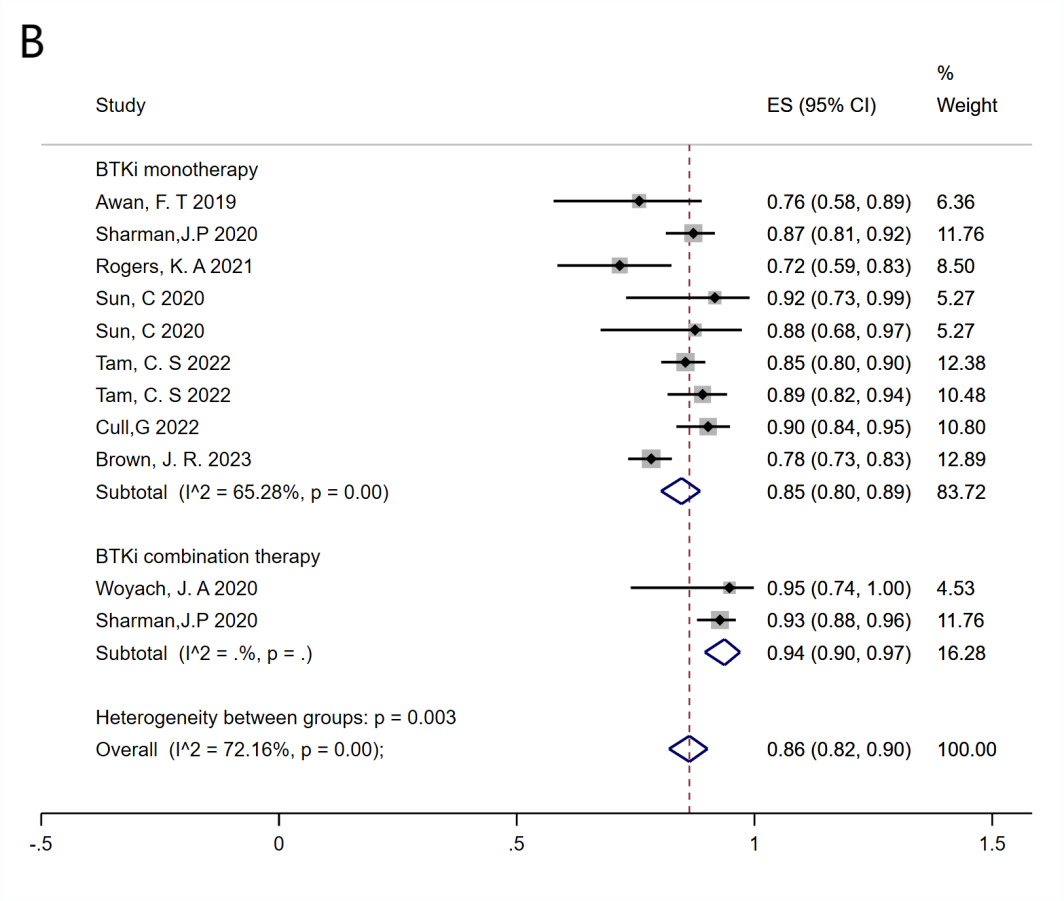


**Figure S9.** Forest plots assessing the effect of BTKi combination therapy vs BTKi monotherapy (A) 24-months OS; (B) 24-months PFS.


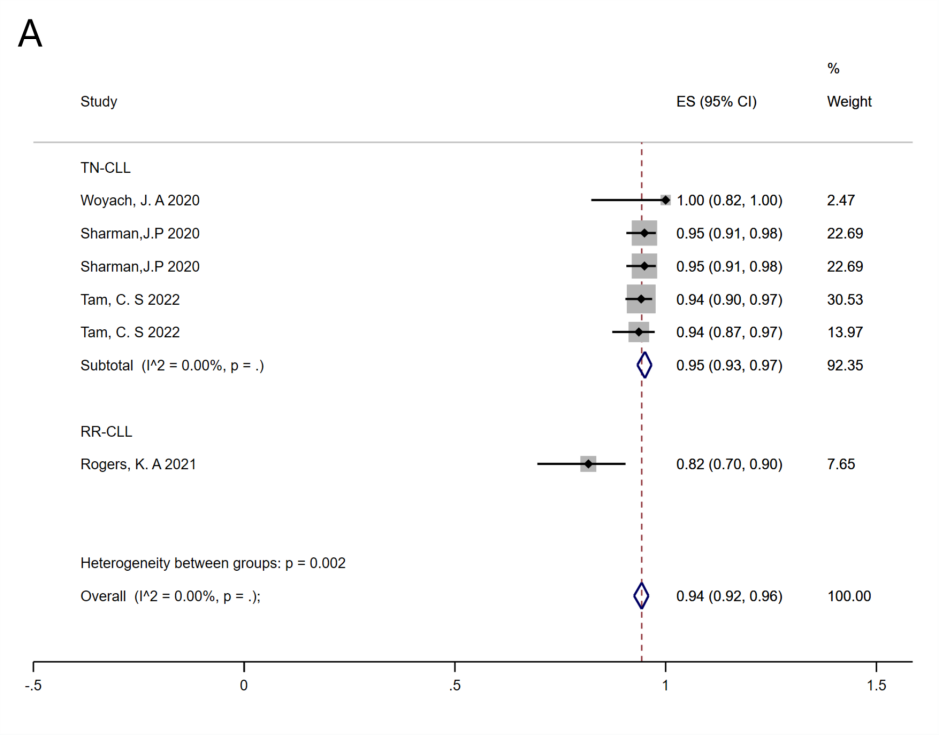


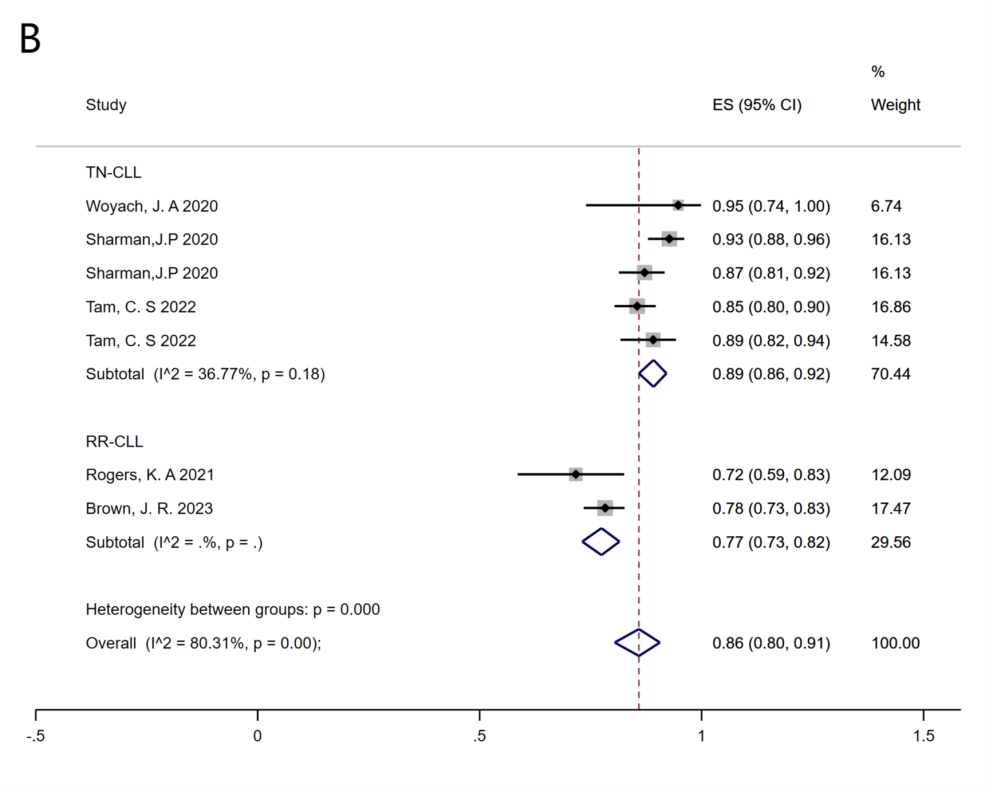


**Figure S10.** Forest plots assessing the effect of disease status (TN-CLL vs RR-CLL) (A) 24-months OS; (B) 24-months PFS.


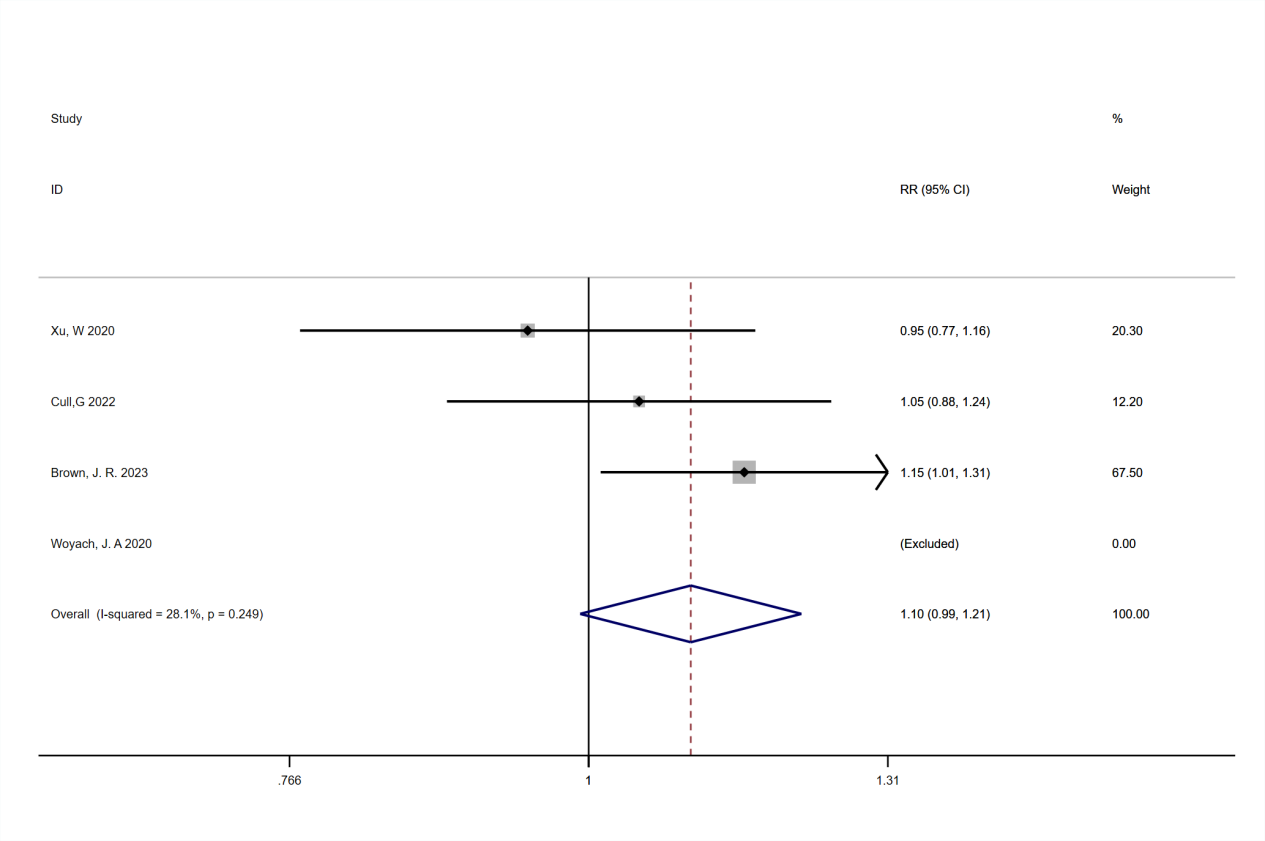


**Figure S11.** Forest plot of the ORR for treatment with the unmutated IGHV vs. mutated IGHV (fixed effect model). RR is the effect size.


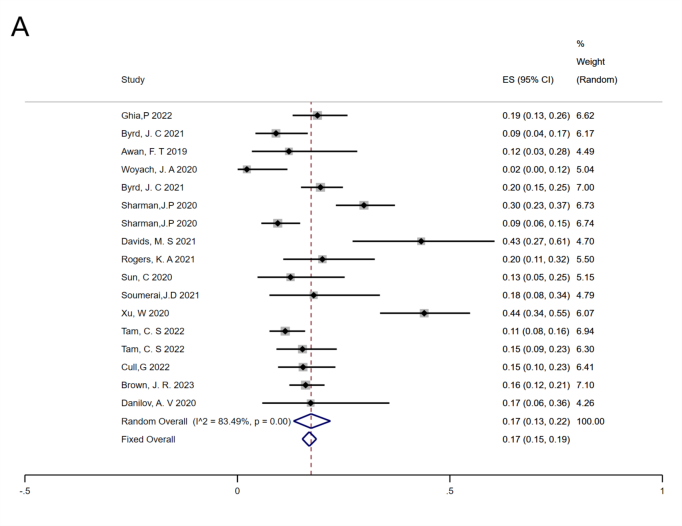

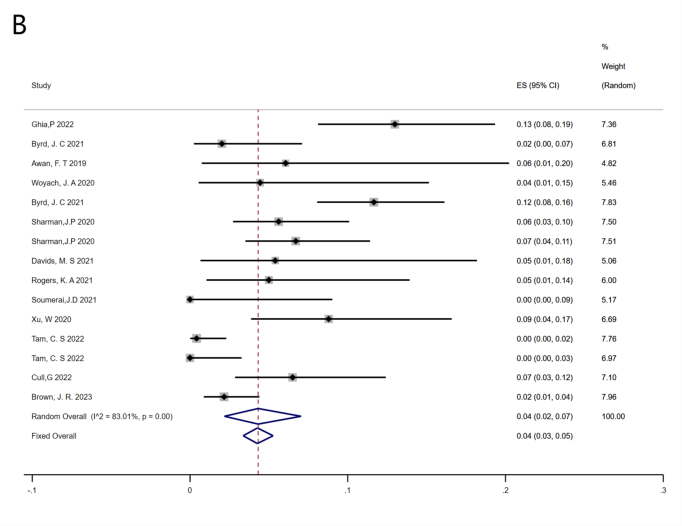

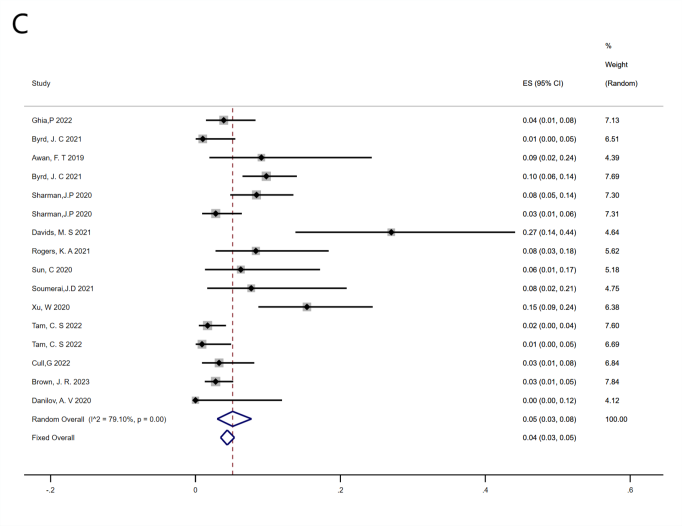

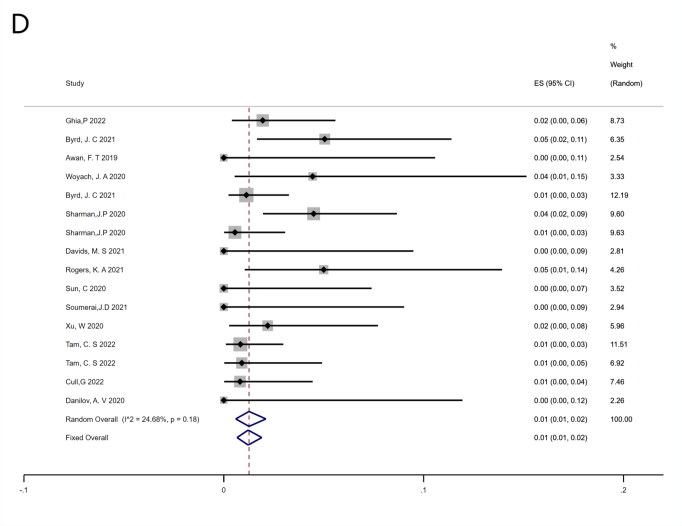


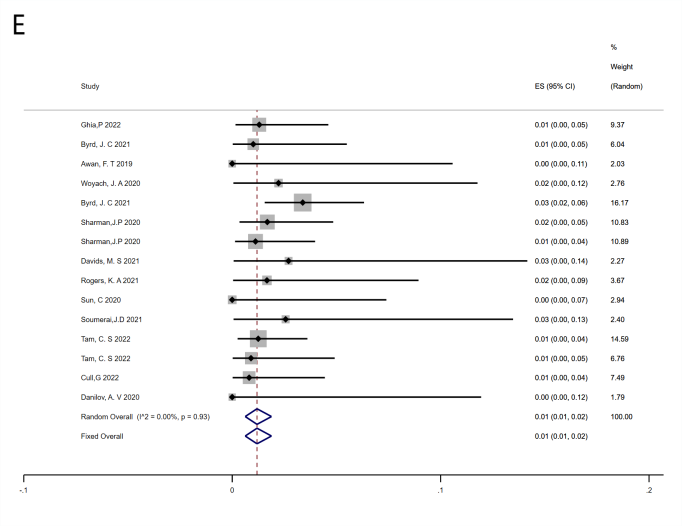

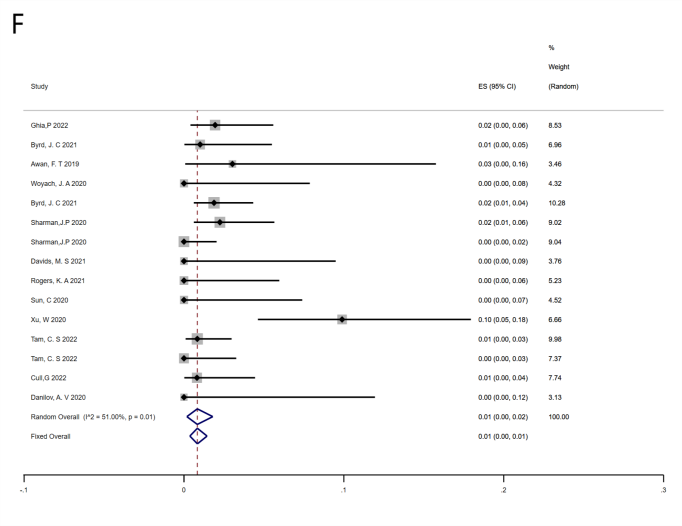


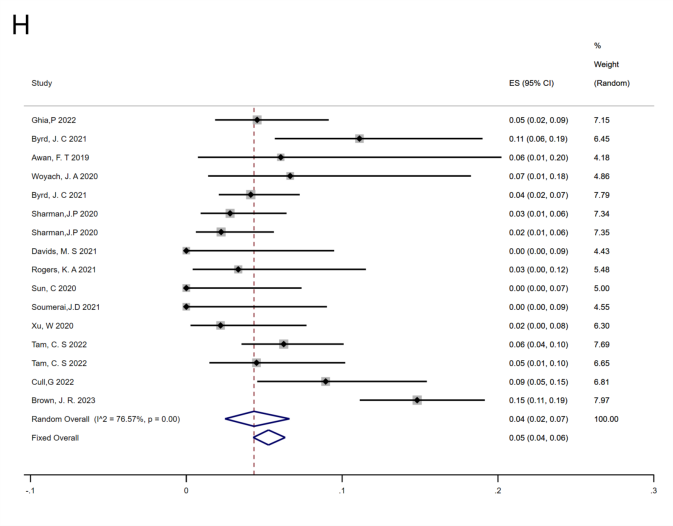

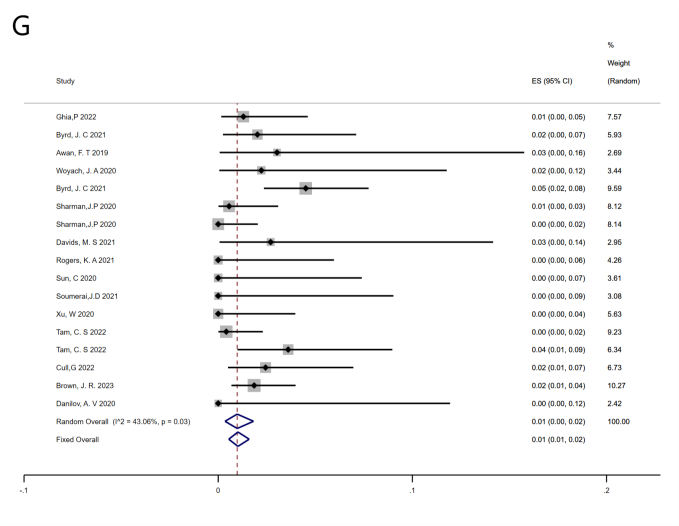


**Figure S12.** Forest plots for pooled grade ≥3 (A) neutropenia; (B) anemia; (C) thrombocytopenia; (D) diarrhea; (E) fatigue; (F) upper respiratory tract infection; (G) atrial fibrillation; (H) hypertension.
