# Supplementary material for: Efficacy and safety of new-generation Bruton tyrosine kinase inhibitors in chronic lymphocytic leukemia/small lymphocytic lymphoma: a systematic review and meta-analysis
Source: Ann Hematol. 2023 Oct 16;103(7):2231–44. doi: 10.1007/s00277-023-05486-x (PMC11224099; doi:10.1007/s00277-023-05486-x)
Supplement: Supplementary file 2 — Supplementary file2 (DOCX 21 KB) [file 277_2023_5486_MOESM2_ESM.docx]

**Table S1.** Search algorithm

| **Search words** | |
| --- | --- |
| **subject word** | **free word** |
| Bruton Tyrosine Kinase Inhibitor | BTK inhibitors; BTK inhibitor; Bruton's Tyrosine Kinase Inhibitors; Bruton Tyrosine Kinase Inhibitors; Bruton's Tyrosine Kinase Inhibitor; inhibitors of Bruton's tyrosine kinase; inhibitors of Bruton tyrosine kinase; inhibitor of Bruton's tyrosine kinase; inhibitor of Bruton tyrosine kinase; Bruton's tyrosine kinase (BTK) inhibitors; Bruton tyrosine kinase (BTK) inhibitors; Bruton's tyrosine kinase (BTK) inhibitor; Bruton tyrosine kinase (BTK) inhibitor; Inhibitors targeting Bruton's tyrosine kinase; Inhibitors targeting Bruton tyrosine kinase; Inhibitor targeting Bruton's tyrosine kinase; Inhibitor targeting Bruton tyrosine kinase; Inhibitors for Bruton's tyrosine kinase; Inhibitors for Bruton tyrosine kinase; Inhibitor for Bruton's tyrosine kinase; Inhibitor for Bruton tyrosine kinase; BTKi |
| zanubrutinib | (7S)-2-(4-phenoxyphenyl)-7-(1-(prop-2-enoyl)piperidin-4-yl)-4,5,6,7-tetrahydropyrazolo(1,5-a)pyrimidine-3-carboxamide; 7-(1-acryloyl-4-piperidinyl)-2-(4-phenoxyphenyl)-4,5,6,7-tetrahydropyrazolo(1,5-a)pyrimidine-3-carboxamide; Brukinsa; BGB-3111 |
| acalabrutinib | Calquence; ACP-196 |
| tirabrutinib | 6-Amino-9-((3R)-1-(2-butynoyl)-3-pyrrolidinyl)-7-(4-phenoxyphenyl)-7,9-dihydro-8H-purin-8-one;8H-Purin-8-one,6-amino-7,9-dihydro-9-((3R)-1-(1-oxo-2-butyn-1-yl)-3-pyrrolidinyl)-7-(4-phenoxyphenyl)-; GS-4059; ONO-4059 |
| orelabrutinib | ICP-002 |
| Leukemia, Lymphocytic, Chronic, B-Cell | Lymphoma, Lymphocytic, Well Differentiated; Lymphoma, Lymphocytic, Well-Differentiated; Lymphoma, Lymphoplasmacytoid, CLL; Lymphoma, Small Lymphocytic; Lymphocytic Lymphoma, Small; Lymphocytic Lymphomas, Small; Lymphomas, Small Lymphocytic; Small Lymphocytic Lymphoma; Small Lymphocytic Lymphomas; Lymphoma, Small Lymphocytic, Plasmacytoid; Lymphoma, Small-Cell; Lymphoma, Small Cell; Lymphomas, Small-Cell; Small-Cell Lymphomas; Lymphoplasmacytoid Lymphoma, CLL; CLL Lymphoplasmacytoid Lymphoma; CLL Lymphoplasmacytoid Lymphomas; Lymphoma, CLL Lymphoplasmacytoid; Lymphomas, CLL Lymphoplasmacytoid; Lymphoplasmacytoid Lymphomas, CLL; Small-Cell Lymphoma; Small Cell Lymphoma  Lymphoblastic Leukemia, Chronic; Chronic Lymphoblastic Leukemia; Chronic Lymphoblastic Leukemias; Leukemias, Chronic Lymphoblastic; Lymphoblastic Leukemias, Chronic; Lymphocytic Leukemia, Chronic; Chronic Lymphocytic Leukemias; Lymphocytic Leukemias, Chronic; Lymphocytic Leukemia, Chronic, B Cell; Lymphocytic Leukemia, Chronic, B-Cell; Lymphocytic Lymphoma; Lymphocytic Lymphomas; Lymphomas, Lymphocytic; Lymphocytic Lymphoma, Diffuse, Well Differentiated; Lymphocytic Lymphoma, Diffuse, Well-Differentiated; Lymphocytic Lymphoma, Well Differentiated; Lymphocytic Lymphoma, Well-Differentiated; Lymphocytic Lymphomas, Well-Differentiated; Lymphoma, Well-Differentiated Lymphocytic; Lymphomas, Well-Differentiated Lymphocytic; Well-Differentiated Lymphocytic Lymphoma; Well-Differentiated Lymphocytic Lymphomas; Lymphoma, Lymphocytic; Lymphoma, Lymphocytic, Diffuse, Well Differentiated; B-Cell Chronic Lymphocytic Leukemia; B Cell Chronic Lymphocytic Leukemia; B-Cell Leukemia, Chronic; B Cell Leukemia, Chronic; B-Cell Leukemias, Chronic; Chronic B-Cell Leukemia; Chronic B-Cell Leukemias; Leukemia, Chronic B-Cell; Leukemias, Chronic B-Cell; B-Cell Malignancy, Low-Grade; B Cell Malignancy, Low Grade; B-Cell Malignancies, Low-Grade; Low-Grade B-Cell Malignancies; Low-Grade B-Cell Malignancy; Malignancies, Low-Grade B-Cell; Malignancy, Low-Grade B-Cell; B-Lymphocytic Leukemia, Chronic; B Lymphocytic Leukemia, Chronic; B-Lymphocytic Leukemias, Chronic; Chronic B-Lymphocytic Leukemia; Chronic B-Lymphocytic Leukemias; Leukemia, Chronic B-Lymphocytic; Leukemias, Chronic B-Lymphocytic; Chronic Lymphocytic Leukemia; Diffuse Well-Differentiated Lymphocytic Lymphoma; Diffuse Well Differentiated Lymphocytic Lymphoma; Disrupted In B-Cell Malignancy; Disrupted In B Cell Malignancy; Leukemia, B Cell, Chronic; Leukemia, B-Cell, Chronic; Leukemia, Chronic Lymphatic; Chronic Lymphatic Leukemia; Chronic Lymphatic Leukemias; Leukemias, Chronic Lymphatic; Lymphatic Leukemia, Chronic; Lymphatic Leukemias, Chronic; Leukemia, Chronic Lymphocytic; Leukemia, Chronic Lymphocytic, B-Cell; Leukemia, Lymphoblastic, Chronic; Leukemia, Lymphocytic, Chronic; Leukemia, Lymphocytic, Chronic, B Cell; Lymphoma, Lymphocytic, Diffuse, Well-Differentiated; CLL; SLL |

| **The search strategy for PubMed** |
| --- |
| ((((((((((((((((((((((((((((((((((((((((((((((((((((((((((((((((((((((((((((((((((((((((((((Lymphoma, Lymphocytic, Well Differentiated[Title/Abstract]) OR (Lymphoma, Lymphocytic, Well-Differentiated[Title/Abstract])) OR (Lymphoma, Lymphoplasmacytoid, CLL[Title/Abstract])) OR (Lymphoma, Small Lymphocytic[Title/Abstract])) OR (Lymphocytic Lymphoma, Small[Title/Abstract])) OR (Lymphocytic Lymphomas, Small[Title/Abstract])) OR (Lymphomas, Small Lymphocytic[Title/Abstract])) OR (Small Lymphocytic Lymphoma[Title/Abstract])) OR (Small Lymphocytic Lymphomas[Title/Abstract])) OR (Lymphoma, Small Lymphocytic, Plasmacytoid[Title/Abstract])) OR (Lymphoma, Small-Cell[Title/Abstract])) OR (Lymphoma, Small Cell[Title/Abstract])) OR (Lymphomas, Small-Cell[Title/Abstract])) OR (Small-Cell Lymphomas[Title/Abstract])) OR (Lymphoplasmacytoid Lymphoma, CLL[Title/Abstract])) OR (CLL Lymphoplasmacytoid Lymphoma[Title/Abstract])) OR (CLL Lymphoplasmacytoid Lymphomas[Title/Abstract])) OR (Lymphoma, CLL Lymphoplasmacytoid[Title/Abstract])) OR (Lymphomas, CLL Lymphoplasmacytoid[Title/Abstract])) OR (Lymphoplasmacytoid Lymphomas, CLL[Title/Abstract])) OR (Small-Cell Lymphoma[Title/Abstract])) OR (Small Cell Lymphoma[Title/Abstract])) OR (Lymphoblastic Leukemia, Chronic[Title/Abstract])) OR (Chronic Lymphoblastic Leukemia[Title/Abstract])) OR (Chronic Lymphoblastic Leukemias[Title/Abstract])) OR (Leukemias, Chronic Lymphoblastic[Title/Abstract])) OR (Lymphoblastic Leukemias, Chronic[Title/Abstract])) OR (Lymphocytic Leukemia, Chronic[Title/Abstract])) OR (Chronic Lymphocytic Leukemias[Title/Abstract])) OR (Lymphocytic Leukemias, Chronic[Title/Abstract])) OR (Lymphocytic Leukemia, Chronic, B Cell[Title/Abstract])) OR (Lymphocytic Leukemia, Chronic, B-Cell[Title/Abstract])) OR (Lymphocytic Lymphoma[Title/Abstract])) OR (Lymphocytic Lymphomas[Title/Abstract])) OR (Lymphomas, Lymphocytic[Title/Abstract])) OR (Lymphocytic Lymphoma, Diffuse, Well Differentiated[Title/Abstract])) OR (Lymphocytic Lymphoma, Diffuse, Well-Differentiated[Title/Abstract])) OR (Lymphocytic Lymphoma, Well Differentiated[Title/Abstract])) OR (Lymphocytic Lymphoma, Well-Differentiated[Title/Abstract])) OR (Lymphocytic Lymphomas, Well-Differentiated[Title/Abstract])) OR (Lymphoma, Well-Differentiated Lymphocytic[Title/Abstract])) OR (Lymphomas, Well-Differentiated Lymphocytic[Title/Abstract])) OR (Well-Differentiated Lymphocytic Lymphoma[Title/Abstract])) OR (Well-Differentiated Lymphocytic Lymphomas[Title/Abstract])) OR (Lymphoma, Lymphocytic[Title/Abstract])) OR (Lymphoma, Lymphocytic, Diffuse, Well Differentiated[Title/Abstract])) OR (B-Cell Chronic Lymphocytic Leukemia[Title/Abstract])) OR (B Cell Chronic Lymphocytic Leukemia[Title/Abstract])) OR (B-Cell Leukemia, Chronic[Title/Abstract])) OR (B Cell Leukemia, Chronic[Title/Abstract])) OR (B-Cell Leukemias, Chronic[Title/Abstract])) OR (Chronic B-Cell Leukemia[Title/Abstract])) OR (Chronic B-Cell Leukemias[Title/Abstract])) OR (Leukemia, Chronic B-Cell[Title/Abstract])) OR (Leukemias, Chronic B-Cell[Title/Abstract])) OR (B-Cell Malignancy, Low-Grade[Title/Abstract])) OR (B Cell Malignancy, Low Grade[Title/Abstract])) OR (B-Cell Malignancies, Low-Grade[Title/Abstract])) OR (Low-Grade B-Cell Malignancies[Title/Abstract])) OR (Low-Grade B-Cell Malignancy[Title/Abstract])) OR (Malignancies, Low-Grade B-Cell[Title/Abstract])) OR (Malignancy, Low-Grade B-Cell[Title/Abstract])) OR (B-Lymphocytic Leukemia, Chronic[Title/Abstract])) OR (B Lymphocytic Leukemia, Chronic[Title/Abstract])) OR (B-Lymphocytic Leukemias, Chronic[Title/Abstract])) OR (Chronic B-Lymphocytic Leukemia[Title/Abstract])) OR (Chronic B-Lymphocytic Leukemias[Title/Abstract])) OR (Leukemia, Chronic B-Lymphocytic[Title/Abstract])) OR (Leukemias, Chronic B-Lymphocytic[Title/Abstract])) OR (Chronic Lymphocytic Leukemia[Title/Abstract])) OR (Diffuse Well-Differentiated Lymphocytic Lymphoma[Title/Abstract])) OR (Diffuse Well Differentiated Lymphocytic Lymphoma[Title/Abstract])) OR (Disrupted In B-Cell Malignancy[Title/Abstract])) OR (Disrupted In B Cell Malignancy[Title/Abstract])) OR (Leukemia, B Cell, Chronic[Title/Abstract])) OR (Leukemia, B-Cell, Chronic[Title/Abstract])) OR (Leukemia, Chronic Lymphatic[Title/Abstract])) OR (Chronic Lymphatic Leukemia[Title/Abstract])) OR (Chronic Lymphatic Leukemias[Title/Abstract])) OR (Leukemias, Chronic Lymphatic[Title/Abstract])) OR (Lymphatic Leukemia, Chronic[Title/Abstract])) OR (Lymphatic Leukemias, Chronic[Title/Abstract])) OR (Leukemia, Chronic Lymphocytic[Title/Abstract])) OR (Leukemia, Chronic Lymphocytic, B-Cell[Title/Abstract])) OR (Leukemia, Lymphoblastic, Chronic[Title/Abstract])) OR (Leukemia, Lymphocytic, Chronic[Title/Abstract])) OR (Leukemia, Lymphocytic, Chronic, B Cell[Title/Abstract])) OR (Lymphoma, Lymphocytic, Diffuse, Well-Differentiated[Title/Abstract])) OR (CLL[Title/Abstract]))) OR (SLL[Title/Abstract])) OR ("Leukemia, Lymphocytic, Chronic, B-Cell"[Mesh])) AND (((((((((((((((((((((((((((Bruton Tyrosine Kinase Inhibitor[Title/Abstract]) OR (BTK inhibitors[Title/Abstract])) OR (BTK inhibitor[Title/Abstract])) OR (Bruton's Tyrosine Kinase Inhibitors[Title/Abstract])) OR (Bruton Tyrosine Kinase Inhibitors[Title/Abstract])) OR (Bruton's Tyrosine Kinase Inhibitor[Title/Abstract])) OR (inhibitors of Bruton's tyrosine kinase[Title/Abstract])) OR (inhibitors of Bruton tyrosine kinase[Title/Abstract])) OR (inhibitor of Bruton's tyrosine kinase[Title/Abstract])) OR (inhibitor of Bruton tyrosine kinase[Title/Abstract])) OR (Bruton's tyrosine kinase (BTK) inhibitors[Title/Abstract])) OR (Bruton tyrosine kinase (BTK) inhibitors[Title/Abstract])) OR (Bruton's tyrosine kinase (BTK) inhibitor[Title/Abstract])) OR (Bruton tyrosine kinase (BTK) inhibitor[Title/Abstract])) OR (Inhibitors targeting Bruton's tyrosine kinase[Title/Abstract])) OR (Inhibitors targeting Bruton tyrosine kinase[Title/Abstract])) OR (Inhibitor targeting Bruton's tyrosine kinase[Title/Abstract])) OR (Inhibitor targeting Bruton tyrosine kinase[Title/Abstract])) OR (Inhibitors for Bruton's tyrosine kinase[Title/Abstract])) OR (Inhibitors for Bruton tyrosine kinase[Title/Abstract])) OR (Inhibitor for Bruton's tyrosine kinase[Title/Abstract])) OR (Inhibitor for Bruton tyrosine kinase[Title/Abstract])) OR (BTKi[Title/Abstract])) OR (((((zanubrutinib[Title/Abstract]) OR ((7S)-2-(4-phenoxyphenyl)-7-(1-(prop-2-enoyl)piperidin-4-yl)-4,5,6,7-tetrahydropyrazolo(1,5-a)pyrimidine-3-carboxamide[Title/Abstract])) OR (Brukinsa[Title/Abstract])) OR (7-(1-acryloyl-4-piperidinyl)-2-(4-phenoxyphenyl)-4,5,6,7-tetrahydropyrazolo(1,5-a)pyrimidine-3-carboxamide[Title/Abstract])) OR (BGB-3111[Title/Abstract]))) OR (((acalabrutinib[Title/Abstract]) OR (Calquence[Title/Abstract])) OR (ACP-196[Title/Abstract]))) OR (((((tirabrutinib[Title/Abstract]) OR (6-Amino-9-((3R)-1-(2-butynoyl)-3-pyrrolidinyl)-7-(4-phenoxyphenyl)-7,9-dihydro-8H-purin-8-one[Title/Abstract])) OR (8H-Purin-8-one, 6-amino-7,9-dihydro-9-((3R)-1-(1-oxo-2-butyn-1-yl)-3-pyrrolidinyl)-7-(4-phenoxyphenyl)-[Title/Abstract])) OR (GS-4059[Title/Abstract])) OR (ONO-4059[Title/Abstract]))) OR ((orelabrutinib[Title/Abstract]) OR (ICP-002[Title/Abstract]))) |

| **The search strategy for Embase** | |
| --- | --- |
| **No.** | **Query** |
| #18 | #3 AND #17 |
| #17 | #4 OR #7 OR #10 OR #13 OR #16 |
| #16 | #14 OR #15 |
| #15 | 'icp-002':ab,ti |
| #14 | orelabrutinib |
| #13 | #11 OR #12 |
| #12 | '6-amino-9-((3r)-1-(2-butynoyl)-3-pyrrolidinyl)-7-(4-phenoxyphenyl)-7,9-dihydro-8h-purin-8-one':ab,ti OR '8h-purin-8-one, 6-amino-7,9-dihydro-9-((3r)-1-(1-oxo-2-butyn-1-yl)-3-pyrrolidinyl)-7-(4-phenoxyphenyl)-':ab,ti OR 'gs-4059':ab,ti OR 'ono-4059':ab,ti OR 'tirabrutinib':ab,ti |
| #11 | tirabrutinib |
| #10 | #8 OR #9 |
| #9 | 'calquence':ab,ti OR 'acp-196':ab,ti |
| #8 | acalabrutinib |
| #7 | #5 OR #6 |
| #6 | '(7s)-2-(4-phenoxyphenyl)-7-(1-(prop-2-enoyl)piperidin-4-yl)-4,5,6,7-tetrahydropyrazolo(1,5-a)pyrimidine-3-carboxamide':ab,ti OR 'brukinsa':ab,ti OR '7-(1-acryloyl-4-piperidinyl)-2-(4-phenoxyphenyl)-4,5,6,7-tetrahydropyrazolo(1,5-a)pyrimidine-3-carboxamide':ab,ti OR 'bgb-3111':ab,ti |
| #5 | zanubrutinib |
| #4 | 'btk inhibitors':ab,ti OR 'btk inhibitor':ab,ti OR 'bruton tyrosine kinase inhibitors':ab,ti OR 'inhibitors of bruton tyrosine kinase':ab,ti OR 'inhibitor of bruton tyrosine kinase':ab,ti OR 'bruton tyrosine kinase (btk) inhibitors':ab,ti OR 'bruton tyrosine kinase (btk) inhibitor':ab,ti OR 'inhibitors targeting bruton tyrosine kinase':ab,ti OR 'inhibitor targeting bruton tyrosine kinase':ab,ti OR 'inhibitors for bruton tyrosine kinase':ab,ti OR 'inhibitor for bruton tyrosine kinase':ab,ti |
| #3 | #1 OR #2 |
| #2 | 'lymphoma, lymphocytic, well differentiated':ab,ti OR 'lymphoma, lymphocytic, well-differentiated':ab,ti OR 'lymphoma, lymphoplasmacytoid, cll':ab,ti OR 'lymphoma, small lymphocytic':ab,ti OR 'lymphocytic lymphoma, small':ab,ti OR 'lymphocytic lymphomas, small':ab,ti OR 'lymphomas, small lymphocytic':ab,ti OR 'small lymphocytic lymphoma':ab,ti OR 'small lymphocytic lymphomas':ab,ti OR 'lymphoma, small lymphocytic, plasmacytoid':ab,ti OR 'lymphoma, small-cell':ab,ti OR 'lymphoma, small cell':ab,ti OR 'lymphomas, small-cell':ab,ti OR 'small-cell lymphomas':ab,ti OR 'lymphoplasmacytoid lymphoma, cll':ab,ti OR 'cll lymphoplasmacytoid lymphoma':ab,ti OR 'cll lymphoplasmacytoid lymphomas':ab,ti OR 'lymphoma, cll lymphoplasmacytoid':ab,ti OR 'lymphomas, cll lymphoplasmacytoid':ab,ti OR 'lymphoplasmacytoid lymphomas, cll':ab,ti OR 'small-cell lymphoma':ab,ti OR 'small cell lymphoma':ab,ti OR 'lymphoblastic leukemia, chronic':ab,ti OR 'chronic lymphoblastic leukemia':ab,ti OR 'chronic lymphoblastic leukemias':ab,ti OR 'leukemias, chronic lymphoblastic':ab,ti OR 'lymphoblastic leukemias, chronic':ab,ti OR 'lymphocytic leukemia, chronic':ab,ti OR 'chronic lymphocytic leukemias':ab,ti OR 'lymphocytic leukemias, chronic':ab,ti OR 'lymphocytic leukemia, chronic, b cell':ab,ti OR 'lymphocytic leukemia, chronic, b-cell':ab,ti OR 'lymphocytic lymphoma':ab,ti OR 'lymphocytic lymphomas':ab,ti OR 'lymphomas, lymphocytic':ab,ti OR 'lymphocytic lymphoma, diffuse, well differentiated':ab,ti OR 'lymphocytic lymphoma, diffuse, well-differentiated':ab,ti OR 'lymphocytic lymphoma, well differentiated':ab,ti OR 'lymphocytic lymphoma, well-differentiated':ab,ti OR 'lymphocytic lymphomas, well-differentiated':ab,ti OR 'lymphoma, well-differentiated lymphocytic':ab,ti OR 'lymphomas, well-differentiated lymphocytic':ab,ti OR 'well-differentiated lymphocytic lymphoma':ab,ti OR 'well-differentiated lymphocytic lymphomas':ab,ti OR 'lymphoma, lymphocytic':ab,ti OR 'lymphoma, lymphocytic, diffuse, well differentiated':ab,ti OR 'b-cell chronic lymphocytic leukemia':ab,ti OR 'b cell chronic lymphocytic leukemia':ab,ti OR 'b-cell leukemia, chronic':ab,ti OR 'b cell leukemia, chronic':ab,ti OR 'b-cell leukemias, chronic':ab,ti OR 'chronic b-cell leukemia':ab,ti OR 'chronic b-cell leukemias':ab,ti OR 'leukemia, chronic b-cell':ab,ti OR 'leukemias, chronic b-cell':ab,ti OR 'b-cell malignancy, low-grade':ab,ti OR 'b cell malignancy, low grade':ab,ti OR 'b-cell malignancies, low-grade':ab,ti OR 'low-grade b-cell malignancies':ab,ti OR 'low-grade b-cell malignancy':ab,ti OR 'malignancies, low-grade b-cell':ab,ti OR 'malignancy, low-grade b-cell':ab,ti OR 'b-lymphocytic leukemia, chronic':ab,ti OR 'b lymphocytic leukemia, chronic':ab,ti OR 'b-lymphocytic leukemias, chronic':ab,ti OR 'chronic b-lymphocytic leukemia':ab,ti OR 'chronic b-lymphocytic leukemias':ab,ti OR 'leukemia, chronic b-lymphocytic':ab,ti OR 'leukemias, chronic b-lymphocytic':ab,ti OR 'chronic lymphocytic leukemia':ab,ti OR 'diffuse well-differentiated lymphocytic lymphoma':ab,ti OR 'diffuse well differentiated lymphocytic lymphoma':ab,ti OR 'disrupted in b-cell malignancy':ab,ti OR 'disrupted in b cell malignancy':ab,ti OR 'leukemia, b cell, chronic':ab,ti OR 'leukemia, b-cell, chronic':ab,ti OR 'leukemia, chronic lymphatic':ab,ti OR 'chronic lymphatic leukemia':ab,ti OR 'chronic lymphatic leukemias':ab,ti OR 'leukemias, chronic lymphatic':ab,ti OR 'lymphatic leukemia, chronic':ab,ti OR 'lymphatic leukemias, chronic':ab,ti OR 'leukemia, chronic lymphocytic':ab,ti OR 'leukemia, chronic lymphocytic, b-cell':ab,ti OR 'leukemia, lymphoblastic, chronic':ab,ti OR 'leukemia, lymphocytic, chronic':ab,ti OR 'leukemia, lymphocytic, chronic, b cell':ab,ti OR 'lymphoma, lymphocytic, diffuse, well-differentiated':ab,ti OR 'cll':ab,ti OR 'sll':ab,ti |
| #1 | ('leukemia,'/exp OR leukemia,) AND lymphocytic, AND chronic, AND ('b cell'/exp OR 'b cell') |

| **The search strategy for Cochrane Library** | |
| --- | --- |
| #1 | (Lymphoma, Lymphocytic, Well Differentiated):ab,ti,kw OR (Lymphoma, Lymphocytic, Well-Differentiated):ab,ti,kw OR (Lymphoma, Lymphoplasmacytoid, CLL):ab,ti,kw OR (Lymphoma, Small Lymphocytic):ab,ti,kw OR (Lymphocytic Lymphoma, Small):ab,ti,kw OR (Lymphocytic Lymphomas, Small):ab,ti,kw OR (Lymphomas, Small Lymphocytic):ab,ti,kw OR (Small Lymphocytic Lymphoma):ab,ti,kw OR (Small Lymphocytic Lymphomas):ab,ti,kw OR (Lymphoma, Small Lymphocytic, Plasmacytoid):ab,ti,kw OR (Lymphoma, Small-Cell):ab,ti,kw OR (Lymphoma, Small Cell):ab,ti,kw OR (Lymphomas, Small-Cell):ab,ti,kw OR (Small-Cell Lymphomas):ab,ti,kw OR (Lymphoplasmacytoid Lymphoma, CLL):ab,ti,kw OR (CLL Lymphoplasmacytoid Lymphoma):ab,ti,kw OR (CLL Lymphoplasmacytoid Lymphomas):ab,ti,kw OR (Lymphoma, CLL Lymphoplasmacytoid):ab,ti,kw OR (Lymphomas, CLL Lymphoplasmacytoid):ab,ti,kw OR (Lymphoplasmacytoid Lymphomas, CLL):ab,ti,kw OR (Small-Cell Lymphoma):ab,ti,kw OR (Small Cell Lymphoma):ab,ti,kw OR (Lymphoblastic Leukemia, Chronic):ab,ti,kw OR (Chronic Lymphoblastic Leukemia):ab,ti,kw OR (Chronic Lymphoblastic Leukemias):ab,ti,kw OR (Leukemias, Chronic Lymphoblastic):ab,ti,kw OR (Lymphoblastic Leukemias, Chronic):ab,ti,kw OR (Lymphocytic Leukemia, Chronic):ab,ti,kw OR (Chronic Lymphocytic Leukemias):ab,ti,kw OR (Lymphocytic Leukemias, Chronic):ab,ti,kw OR (Lymphocytic Leukemia, Chronic, B Cell):ab,ti,kw OR (Lymphocytic Leukemia, Chronic, B-Cell):ab,ti,kw OR (Lymphocytic Lymphoma):ab,ti,kw OR (Lymphocytic Lymphomas):ab,ti,kw OR (Lymphomas, Lymphocytic):ab,ti,kw OR (Lymphocytic Lymphoma, Diffuse, Well Differentiated):ab,ti,kw OR (Lymphocytic Lymphoma, Diffuse, Well-Differentiated):ab,ti,kw OR (Lymphocytic Lymphoma, Well Differentiated):ab,ti,kw OR (Lymphocytic Lymphoma, Well-Differentiated):ab,ti,kw OR (Lymphocytic Lymphomas, Well-Differentiated):ab,ti,kw OR (Lymphoma, Well-Differentiated Lymphocytic):ab,ti,kw OR (Lymphomas, Well-Differentiated Lymphocytic):ab,ti,kw OR (Well-Differentiated Lymphocytic Lymphoma):ab,ti,kw OR (Well-Differentiated Lymphocytic Lymphomas):ab,ti,kw OR (Lymphoma, Lymphocytic):ab,ti,kw OR (Lymphoma, Lymphocytic, Diffuse, Well Differentiated):ab,ti,kw OR (B-Cell Chronic Lymphocytic Leukemia):ab,ti,kw OR (B Cell Chronic Lymphocytic Leukemia):ab,ti,kw OR (B-Cell Leukemia, Chronic):ab,ti,kw OR (B Cell Leukemia, Chronic):ab,ti,kw OR (B-Cell Leukemias, Chronic):ab,ti,kw OR (Chronic B-Cell Leukemia):ab,ti,kw OR (Chronic B-Cell Leukemias):ab,ti,kw OR (Leukemia, Chronic B-Cell):ab,ti,kw OR (Leukemias, Chronic B-Cell):ab,ti,kw OR (B-Cell Malignancy, Low-Grade):ab,ti,kw OR (B Cell Malignancy, Low Grade):ab,ti,kw OR (B-Cell Malignancies, Low-Grade):ab,ti,kw OR (Low-Grade B-Cell Malignancies):ab,ti,kw OR (Low-Grade B-Cell Malignancy):ab,ti,kw OR (Malignancies, Low-Grade B-Cell):ab,ti,kw OR (Malignancy, Low-Grade B-Cell):ab,ti,kw OR (B-Lymphocytic Leukemia, Chronic):ab,ti,kw OR (B Lymphocytic Leukemia, Chronic):ab,ti,kw OR (B-Lymphocytic Leukemias, Chronic):ab,ti,kw OR (Chronic B-Lymphocytic Leukemia):ab,ti,kw OR (Chronic B-Lymphocytic Leukemias):ab,ti,kw OR (Leukemia, Chronic B-Lymphocytic):ab,ti,kw OR (Leukemias, Chronic B-Lymphocytic):ab,ti,kw OR (Chronic Lymphocytic Leukemia):ab,ti,kw OR (Diffuse Well-Differentiated Lymphocytic Lymphoma):ab,ti,kw OR (Diffuse Well Differentiated Lymphocytic Lymphoma):ab,ti,kw OR (Disrupted In B-Cell Malignancy):ab,ti,kw OR (Disrupted In B Cell Malignancy):ab,ti,kw OR (Leukemia, B Cell, Chronic):ab,ti,kw OR (Leukemia, B-Cell, Chronic):ab,ti,kw OR (Leukemia, Chronic Lymphatic):ab,ti,kw OR (Chronic Lymphatic Leukemia):ab,ti,kw OR (Chronic Lymphatic Leukemias):ab,ti,kw OR (Leukemias, Chronic Lymphatic):ab,ti,kw OR (Lymphatic Leukemia, Chronic):ab,ti,kw OR (Lymphatic Leukemias, Chronic):ab,ti,kw OR (Leukemia, Chronic Lymphocytic):ab,ti,kw OR (Leukemia, Chronic Lymphocytic, B-Cell):ab,ti,kw OR (Leukemia, Lymphoblastic, Chronic):ab,ti,kw OR (Leukemia, Lymphocytic, Chronic):ab,ti,kw OR (Leukemia, Lymphocytic, Chronic, B Cell):ab,ti,kw OR (Lymphoma, Lymphocytic, Diffuse, Well-Differentiated):ab,ti,kw OR (CLL):ab,ti,kw OR (SLL):ab,ti,kw |
| #2 | Leukemia, Lymphocytic, Chronic, B-Cell |
| #3 | #1 or #2 |
| #4 | (BTK inhibitor):ab,ti,kw OR (BTK inhibitors):ab,ti,kw OR (Bruton's Tyrosine Kinase Inhibitors):ab,ti,kw OR (Bruton Tyrosine Kinase Inhibitors):ab,ti,kw OR (Bruton's Tyrosine Kinase Inhibitor):ab,ti,kw OR (inhibitors of Bruton's tyrosine kinase):ab,ti,kw OR (inhibitors of Bruton tyrosine kinase):ab,ti,kw OR (inhibitor of Bruton's tyrosine kinase):ab,ti,kw OR (inhibitor of Bruton tyrosine kinase):ab,ti,kw OR (Bruton's tyrosine kinase (BTK) inhibitors):ab,ti,kw OR (Bruton tyrosine kinase (BTK) inhibitors):ab,ti,kw OR (Bruton's tyrosine kinase (BTK) inhibitor):ab,ti,kw OR (Bruton tyrosine kinase (BTK) inhibitor):ab,ti,kw OR (Inhibitors targeting Bruton's tyrosine kinase):ab,ti,kw OR (Inhibitors targeting Bruton tyrosine kinase):ab,ti,kw OR (Inhibitor targeting Bruton's tyrosine kinase):ab,ti,kw OR (Inhibitor targeting Bruton tyrosine kinase):ab,ti,kw OR (Inhibitors for Bruton's tyrosine kinase):ab,ti,kw OR (Inhibitors for Bruton tyrosine kinase):ab,ti,kw OR (Inhibitor for Bruton's tyrosine kinase):ab,ti,kw OR (Inhibitor for Bruton tyrosine kinase):ab,ti,kw OR (BTKi):ab,ti,kw |
| #5 | Bruton Tyrosine Kinase Inhibitor |
| #6 | #4 or #5 |
| #7 | acalabrutinib |
| #8 | (Calquence):ab,ti,kw OR (ACP-196):ab,ti,kw |
| #9 | #7 or #8 |
| #10 | zanubrutinib |
| #11 | (Brukinsa):ab,ti,kw OR (BGB-3111):ab,ti,kw |
| #12 | #10 or #11 |
| #13 | tirabrutinib |
| #14 | (GS-4059):ab,ti,kw OR (ONO-4059):ab,ti,kw |
| #15 | #13 or #14 |
| #16 | orelabrutinib |
| #17 | ICP-002 |
| #18 | #16 or #17 |
| #19 | #6 or #9 or #12 or #15 or #18 |
| #20 | #19 and #3 |
